# Drug-Herb Interactions among Thai Herbs and Anticancer Drugs: A Scoping Review

**DOI:** 10.3390/ph15020146

**Published:** 2022-01-26

**Authors:** Apisada Jiso, Phisit Khemawoot, Pinnakarn Techapichetvanich, Sutinee Soopairin, Kittiphong Phoemsap, Panrawee Damrongsakul, Supakit Wongwiwatthananukit, Pornpun Vivithanaporn

**Affiliations:** 1 Chakri Naruebodindra Medical Institute, Faculty of Medicine Ramathibodi Hospital, Mahidol University, Bang Phli, Samut Prakarn 10540, Thailand; jiso.apisada@gmail.com (A.J.); phisit.khe@mahidol.ac.th (P.K.); 2 Program in Translational Medicine, Faculty of Medicine Ramathibodi Hospital, Mahidol University, Bangkok 10400, Thailand; pinnatecha@gmail.com; 3 Department of Biochemistry and Microbiology, Faculty of Pharmaceutical Sciences, Chulalongkorn University, Bangkok 10330, Thailand; sutinee.ssoo@gmail.com (S.S.); Kitiphong.phs@gmail.com (K.P.); pa.damrongsakul@gmail.com (P.D.); 4 Department of Pharmacy Practice, Daniel K. Inouye College of Pharmacy, University of Hawaii at Hilo, Hilo, HI 96720, USA; supakit@hawaii.edu

**Keywords:** drug-herb interactions, anticancer drugs, Thai herbs, tropical herbs

## Abstract

More than half of Thai patients with cancer take herbal preparations while receiving anticancer therapy. There is no systematic or scoping review on interactions between anticancer drugs and Thai herbs, although several research articles have that Thai herbs inhibit cytochrome P450 (CYP) or efflux transporter. Therefore, we gathered and integrated information related to the interactions between anticancer drugs and Thai herbs. Fifty-two anticancer drugs from the 2020 Thailand National List of Essential Medicines and 75 herbs from the 2020 Thai Herbal Pharmacopoeia were selected to determine potential anticancer drug–herb interactions. The pharmacological profiles of the selected anticancer drugs were reviewed and matched with the herbal pharmacological activities to determine possible interactions. A large number of potential anticancer drug–herb interactions were found; the majority involved CYP inhibition. Efflux transporter inhibition and enzyme induction were also found, which could interfere with the pharmacokinetic profiles of anticancer drugs. However, there is limited knowledge on the pharmacodynamic interactions between anticancer drugs and Thai herbs. Therefore, further research is warranted. Information regarding interactions between anticancer drugs and Thai herbs should provide as a useful resource to healthcare professionals in daily practice. It could enable the prediction of possible anticancer drug–herb interactions and could be used to optimize cancer therapy outcomes.

## 1. Introduction

According to the World Health Organization, cancer was one of the top 10 causes of worldwide death in 2019 [1]. In 2020, there were 190,636 new cases of patients with cancer and 124,866 deaths from cancer reported in Thailand [2]. Cancer is a group of diseases caused by an abnormality in cell proliferation and differentiation, which results in an invasion into organs, leading to metastasis and death [3]. All cancer survivors are at risk of cancer recurrence despite receiving effective treatments, as some cancer cells remain in their bodies [4]. Currently, patients with cancer are treated with many types of chemotherapeutic agents, which predispose them to high incidences of adverse drug reactions and put them at high risk of drug–drug interactions, resulting in sub-therapeutic effects or increased unwanted toxicities that could potentiate the negative outcomes of cancer therapy [5]. Moreover, there are reports on herbal medicines used by patients with cancer as an alternative or supportive treatment. In one study, 433 out of 806 patients with cancer used herbal medicines while receiving chemotherapy [6]. Herbal medicine commonly used in European and Middle Eastern countries is associated with the potential risks of cytochrome P450 (CYP) induction or inhibition, altered pharmacodynamics or the reduction of anticancer resistance in *in vitro* models [7,8]. Since patients with cancer often take herbs to prevent and relieve the symptoms and adverse effects from anticancer drugs [9], healthcare professionals should be aware and must be vigilant against anticancer drug–herb interaction (DHI) problems arising from the use of herbs as an alternative or supportive treatment [10,11].

Using tropical herbs as an alternative cancer treatment may cause potential DHI and affect the efficacy and safety of anticancer drugs. Thus, information on anticancer drug–herb interactions could minimize or prevent problems and assist healthcare professionals to educate their patients about DHI. There is no systematic or scoping review available in which researchers have discussed interaction between anticancer drugs and commonly used Thai herbs that are relevant to clinical practice and have identified and searched for potential interactions. Therefore, we developed a scoping review of DHIs by selecting anticancer drugs from the 2020 Thailand National List of Essential Medicines (NLEM) [12] and herbs from the 2020 Thai Herbal Pharmacopoeia (THP) [13]. These herbs, such as turmeric (*Curcuma longa*), garlic (*Allium sativum*), pepper (*Piper nigrum*), and green chiretta (*Andrographis paniculata*), are commonly found in Thailand, China, India and other Southeast Asian countries. This information could be a useful resource to allow healthcare professionals to identify possible anticancer drug–herb interactions and optimize cancer therapy outcomes.

## 2. Results

**Figure 1:**
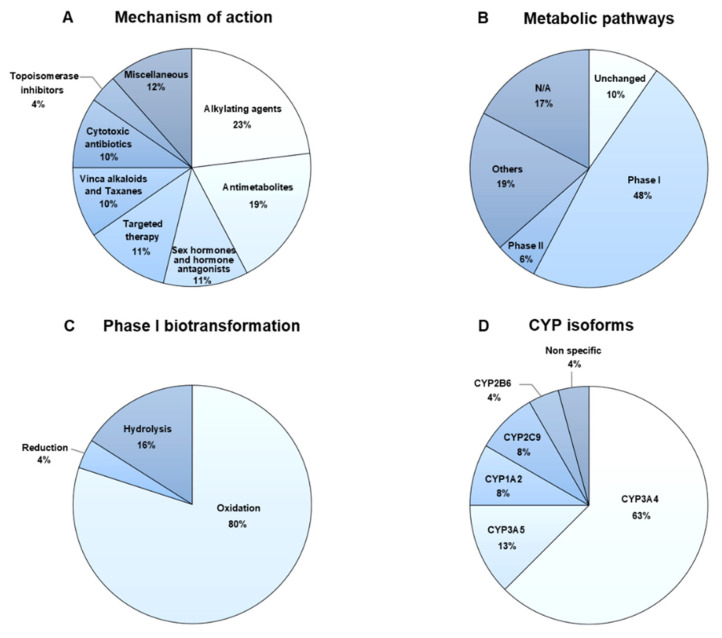
Characteristics of anticancer drugs: (**A**) mechanism of action; (**B**) metabolic pathways; (**C**) phase I biotransformation; and (**D**) cytochrome P450 (CYP) isoforms responsible for metabolism.

The majority of the anticancer drugs in the 2020 NLEM are alkylating agents (23%) and antimetabolites (19%) (Figure 1A). Approximately half of the anticancer drugs are metabolized by phase I biotransformation (Figure 1B). Among phase I metabolism, 80% of anticancer pharmacokinetic profiles involve biotransformation by oxidation, especially via CYP isoforms and, to a lesser degree, by hydrolysis and reduction (Figure 1C). The major enzyme in anticancer metabolism is CYP3A4 (Figure 1D). Several anticancer drugs are excreted via the renal tubules and/or the hepatobiliary system by transmembrane transporters, especially P-glycoprotein. The pharmacokinetic profiles of the selected anticancer drugs are shown in Supporting information (Table S1).

**Figure 2:**
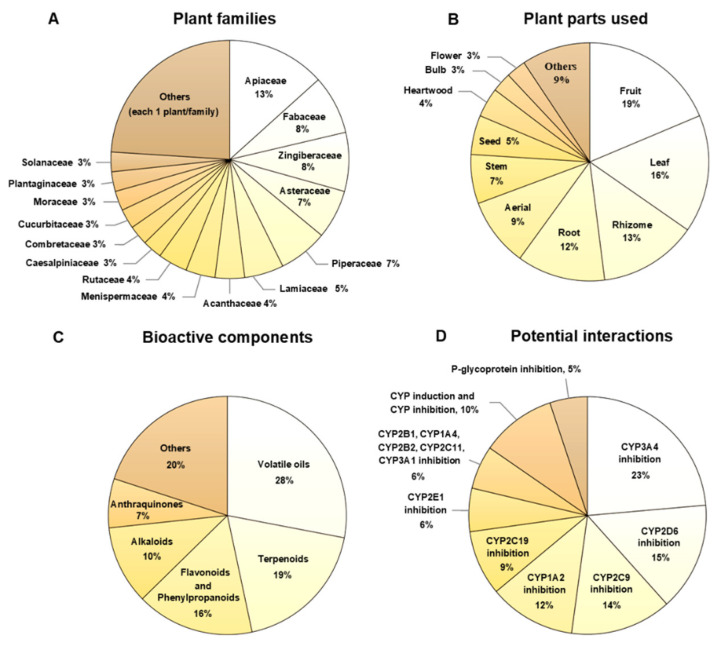
Characteristics of Thai herbs: (**A**) plant families; (**B**) plant parts used; (**C**) bioactive components; and (**D**) potential interactions. Most herbs could inhibit cytochrome P450 (CYP) isoforms and P-glycoprotein; 10% of herbs could inhibit one or more CYP isoform, while inducing other CYP isoforms.

The Thai herbs in the 2020 THP are distributed in 33 families and 13% of them are in the Apiaceae or Umbelliferae family (Figure 2A). Fruits, leaves and rhizomes are common parts that have medicinal properties (Figure 2B). The major bioactive components in these herbs are volatile oils (28%), followed by terpenoids (including triterpenoid saponins, 19%), flavonoids and phenylpropanoids (16%) (Figure 2C). Approximately half of the Thai herbs in the 2020 THP (44%) could alter drug metabolizing enzymatic activities in an *in vitro* setting, especially inhibition of CYP3A4 and CYP2D6. In addition, some Thai herbs could inhibit efflux transporters, particularly P-glycoprotein (Figure 2D).

Among the 52 anticancer drugs and 75 Thai herbs we selected, there are 565 potential anticancer drug–herb interactions. Approximately 90% of these interactions involve CYP inhibition, while some of the interactions exhibit potent CYP inhibitory activity. Potential anticancer drug–herb interactions might occur via drug metabolizing enzymes and efflux transporter inhibition. When categorized by the level of documentation according to the criteria in Table S2, 15 pairs are classified as good and 550 pairs are classified as fair.

All potential interferences with the activities of drug metabolizing enzymes and transporters by Thai herbs are shown in Table 1.

*Andrographis paniculata*, *Centella asiatica*, *Curcuma longa*, *Kaempferia parviflora*, and *Zingiber montanum* are most commonly used in Thai herbal medicine, sometimes referred to as the Thai herbal product champions [106,107]. Our findings have revealed multiple anticancer drugs–herb interactions involving various CYP isoforms and P-glycoprotein transporters. These interactions could have effects on the therapeutic activities and toxicities of anticancer drugs (Table 2).

**Figure 3:**
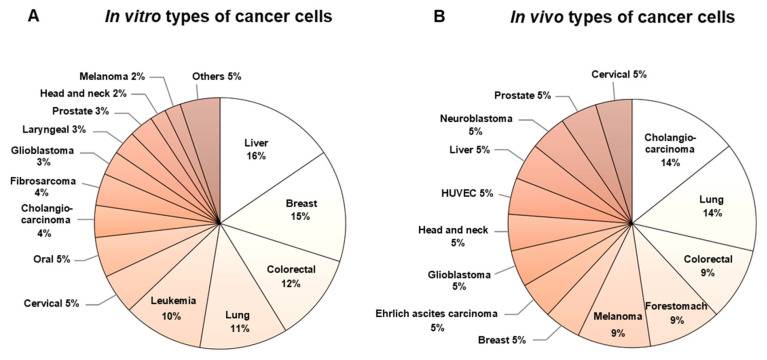
*In vitro* (**A**) and *in vivo* (**B**) experiments of cancer cells used in Thai herbs studies.

Interestingly, many Thai herbs in our study exhibit anticancer activities (Table S3). More than of the half (39 out of 75) have been reported to show cytotoxic effects against cancer cell lines or in *in vivo* models. The most common cell types used in *in vitro* studies have been liver (16%), breast (15%) and colorectal (12%) (Figure 3A), whereas only 16 herbs (21%) have shown anticancer activity in *in vivo* studies. The most reported cell types have been cholangiocarcinoma (14%), lung (14%) and colorectal (9%) (Figure 3B).

## 3. Discussion

Drug-herb interactions could result in therapeutic failure and lead to severe adverse events. One of the most well-known natural products that interferes with drug metabolic pathways is grapefruit juice. Naringin from this citrus fruit inhibits major drug metabolizing enzymes, including CYP3A4 [109]. In our database, piperine in pepper (*Piper nigrum*) also showed strong inhibitory properties against CYP3A4. Therefore, it is possible that the levels of anticancer drugs metabolized mainly by this enzyme would be increased, resulting in more side effects. However, anticancer drugs given as prodrugs (for example, tamoxifen) present decreased efficacy after CYP inhibition due to the reduction in active metabolite [110,111,112,113,114,115,116]. Surprisingly, some of the Thai herbs differentially inhibit several CYP isoforms. For example, *Atractylodes lancea* markedly inhibits CYP1A2 and moderately inhibits CYP2C19, with weak inhibition of CYP2D6 and CYP3A4. This herb may also interfere with the metabolism of several anticancer drugs [117,118]. The majority of DHIs found in this study are related to CYP inhibition [53]. Therefore, the increased levels of anticancer drugs after concomitant use of some herbs and anticancer drugs should be monitored carefully.

Several Thai herbs that are commonly used as food ingredients show CYP inhibitory properties. *Curcuma longa* contains curcuminoids as bioactive ingredients, which have been found to be CYP inhibitors (for example, CYP1A2, CYP2B6, CYP2C9, CYP2C19, CYP2D6, and CYP3A4) [19,65,66]. Thus, anticancer drug–spice interactions should also be a concern for patients with cancer due to the ability of these herbal products to inhibit drug metabolizing enzyme. Curcuminoids have recently been proposed as a bioenhancer for several conventional drugs [119]. Hence, elevated anticancer drug bioavailability and toxicity might occur during the coadministration of *Curcuma longa* and anticancer drugs.

*Centella asiatica*, a major herbal product of Thailand, has a bioactive component consisting of a triterpenoid glycoside and triterpenic acid. This herbal extract has shown mild-to-moderate inhibitory properties against several CYP isoforms, including CYP2C9 and CYP2C19 [38,61,62,120,121]. Moreover, there are reports of increased blood clotting time after the coadministration of *Centella asiatica* with warfarin [122]. Thus, practitioners are aware of and are vigilant of potential toxicities in patients taking *Centella asiatica* with a narrow therapeutic window of drugs metabolized via CYP2C9 or CYP2C19.

*Allium sativum*, commonly called garlic, is a widely used herb and spice in Thailand that affects anticancer drug levels. A clinical study of patients with breast cancer receiving docetaxel as monotherapy showed that the drug clearance was reduced after garlic administration. Moreover, there were genetic polymorphisms associated with the decline in docetaxel clearance [123]. Although the finding did not reach statistical significance due to a small number of participants and possible compensatory metabolic mechanisms of the drug, these findings suggest that coadministration of garlic and docetaxel affect the anticancer drug pharmacokinetics. Further investigation is required to provide clinical evidence of the undesirable adverse effects due to anticancer drug–herb pharmacokinetic interactions.

**Figure 4:**
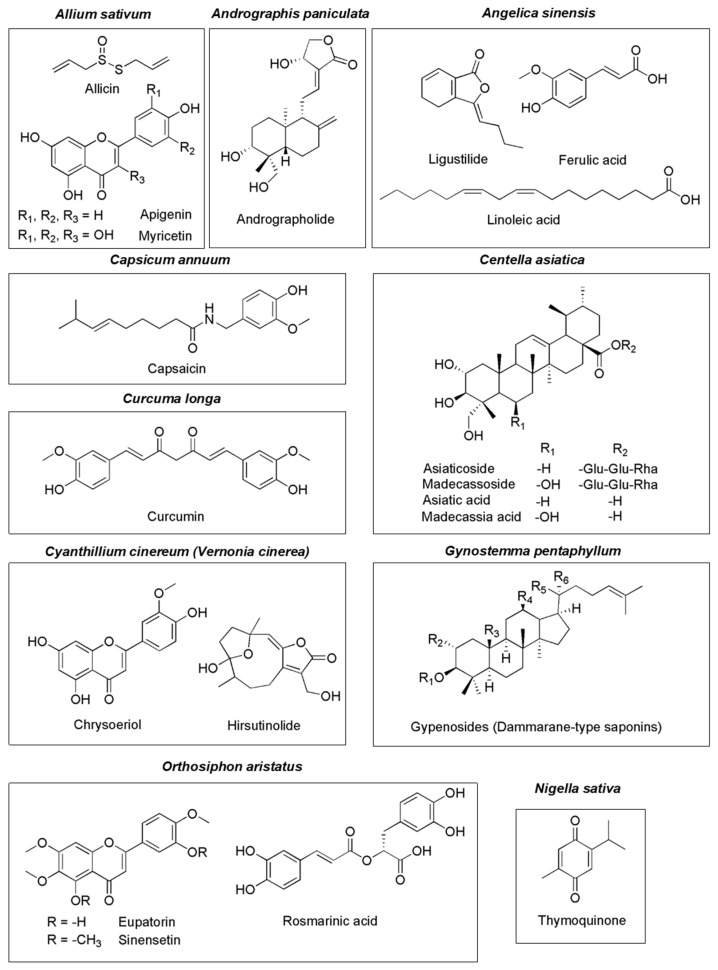
Major compounds found in commonly used Thai herbs.

Considering pharmacodynamic interactions, several herbs in the 2020 THP show anticancer activity. The majority of the reports have focused on *in vitro* apoptotic cell death of cancer cell lines via various mechanisms. In addition, some major Thai herbal products (both pure compounds and extracts) show promising *in vivo* antiproliferative activity. * Andrographis paniculata* extract and andrographolide inhibit tumor-specific angiogenesis by regulating the production of various pro and antiangiogenic factors such as proinflammatory cytokines, nitric oxide, vascular endothelial growth factor (VEGF), interleukin (IL)-2 and tissue inhibitor of metalloproteinase-1 [124,125]. Co-administration of or pre-treatment with pure compounds from tropical herbs such as curcumin from *Curcuma longa*, thymoquinone from *Nigella sativa*, capsaicin from *Capsicum annuum*, or andrographolide from *Andrographis paniculata* together with anticancer drugs enhances anticancer activity via a synergistic effect. There are several common anticancer drugs that show synergistic effects when co-administered with herbs, including fluorouracil, topotecan, paclitaxel, docetaxel, and cisplatin. The interaction effect when curcumin is co-administered with anticancer drugs has reviewed by Tan and Norhaizan [126]. Thymoquinone and topotecan separately arrest the S phase of the cell cycle. The combination of thymoquinone and topotecan increases the amount of fragmented DNA and induces apoptosis through p53- and Bax/Bcl2-independent mechanisms [92]. Capsaicin also enhances *in vitro* and *in vivo* inhibitory effects and induces autophagy of 5-FU and cisplatin [55,59]. The combination of andrographolide and topotecan, gemcitabine, vincristine, cisplatin, arsenic trioxide, and paclitaxel promotes apoptosis in various cancer cell lines [26,28,29,31,36,43,44,45]. The chemical structures of major compounds from commonly used Thai herbs with potential anticancer–herb interactions are shown in Figure 4.

Pharmacodynamic research in the clinical context is needed to determine the anticancer activities of Thai herbs. An evaluation of benefits and risks should be conducted by considering both pharmacokinetic interactions and pharmacodynamics to optimize cancer therapy.

The management of potential DHI between anticancer drugs and Thai herbs seems to be one of the major problems in patient care in some countries, especially in Thailand. Both phytopharmaceutical products and food ingredients from Thai herbs could affect the outcomes of cancer therapies and increase the side effects. Thus, patient education and consultation from healthcare professionals (i.e., physicians or pharmacists) are necessary before the co-administration of anticancer drugs and Thai herbs. The algorithm ‘ask, check and consult’ could increase the safety of the co-administration of anticancer drugs and Thai herbs [127].

This review on interactions between anticancer drugs and Thai herbs provides healthcare professionals with comprehensive information for patient consultation. This study is limited by the number of anticancer drugs: there are only 52 anticancer drugs on the 2020 NLEM. This might not represent all commercially available anticancer drugs Since these are the drugs covered by Thailand’s universal health insurance, and thus they are used extensively. Another limitation is that we considered only 75 herbs derived from the 2020 THP. We did not include mixtures of preparations of several herbs in this study. Further investigation is needed to complete our database of interactions between anticancer drugs and Thai herbs.

## 4. Materials and Methods

### 4.1. Selection of Anticancer Drugs and Herbs

**Figure 5:**
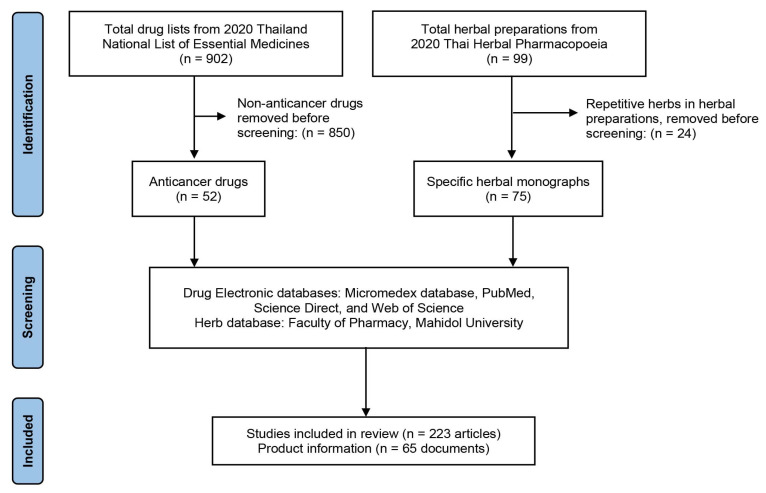
Selection process of anticancer drugs and Thai herbs for the development of DHI information.

Fifty-two anticancer drugs from the 2020 NLEM and 99 Thai herbs from the 2020 THP were selected. Twenty-four herbal items were excluded due to the fact that they were part of herbal preparations (mixtures of multiple herbs). The selection procedure and lists of anticancer drugs and Thai herbs are shown in Figure 5 and Table 3, respectively.

### 4.2. Criteria for the Literature Review

We collected pharmacokinetic, pharmacodynamic, toxicological, and drug interaction data of anticancer drugs by using the Micromedex database, which we accessed under the copyright license of Chulalongkorn University (2020). If the drug data were not available in the database, we used PubMed, Science Direct, and Web of Science to find information on metabolic pathways and drug interactions. For the pharmacologic information on Thai herbs, we used the herb database from the Faculty of Pharmacy, Mahidol University, Thailand, and also available online databases (PubMed, Science Direct, and Web of Science). These data provide the pharmacodynamic activities and the possibility of drug–herb interactions. All data were gathered and analyzed from 1 January to 31 December 2020. The keywords for data collection were:

1. (‘Scientific name of herbs’ OR ‘Common name of herbs’ OR ‘major components of herbs’);
2. (‘*In vitro*’ OR ‘*In vivo*’ OR case reports OR clinical trials);
3. (cytotoxicity OR antiproliferative activity OR anticancer);
4. (Drug-herbs interaction OR Pharmacokinetic OR Pharmacodynamic);
5. (‘anticancer drug name’)

The classification criteria of the severity level and documentation are reported in Table S2. We matched two sets of collected data (anticancer drugs and Thai herbs) and analyzed them individually for potential of anticancer drug–herb interactions. We then evaluated the information on the severity, documentation, and mechanisms of these interactions.

## Figures and Tables

**Table 1:** Potential interactions of drug metabolizing enzyme and transporter activities by Thai herbs.

Thai Herbs	Potential Interactions	References
*Acorus calamus*	- N/A	
*Aegle marmelos*	- CYP3A4 and CYP1A2 inhibition	[14]
*Albizia procera*	- N/A	
*Allium ascalonicum*	- N/A	
*Allium sativum*	- CYP1A, CYP2B, CYP2C, CYP2E1, CYP3A induction - CYP1A2, CYP2C9, CYP2C19, CYP2D6, CYP2E1, CYP3A4, CYP3A5, P-glycoprotein inhibition - Reduce cyclophosphamide-induced developmental toxicity - Interact with tamoxifen	[15,16,17,18,19,20,21,22,23,24,25]
*Andrographis paniculata*	- Potent CYP2A4 and CYP2B9 induction (Andrographolide) - CYP1A2, CYP2B1, CYP2C, CYP2C9, CYP2C19, CYP2C11, CYP2D6, CYP3A, CYP3A1, CYP3A4, UGT1A1, UGT1A3, UGT1A6, UGT1A7, UGT1A8, UGT1A10, UGT2B7, and P-glycoprotein inhibition - Strong synergistic induction of CYP1A1 and CYP1B1 expression (Combination of Andrographolide and CYP1A1 inducers) - Synergistic effects on anticancer activity of 5-FU, arsenic trioxide, bleomycin, carboplatin, cisplatin, doxorubicin, gemcitabine, paclitaxel, topotecan, and vincristine	[26,27,28,29,30,31,32,33,34,35,36,37,38,39,40,41,42,43,44,45]
*Anethum graveolens*	- CYP3A4 inhibition	[19]
*Angelica dahurica*	- N/A	
*Angelica sinensis*	- CYP2D6, CYP3A4, CYP1A2 induction and CYP2E1, CYP3A inhibition	[46,47,48]
*Arcangelisia flava*	- N/A	
*Areca catechu*	- CYP3A4 inhibition	[49]
*Artemisia annua*	- CYP1A1, CYP3A4, moderate CYP1A2, CYP2C19, CYP3A inhibition, and weak CYP2E1inhibition	[49,50,51]
*Atractylodes lancea*	- Potent CYP1A2 inhibition, moderate CYP2E1 and CYP2C19 inhibition, low CYP2D6 and CYP3A4 inhibition	[52,53]
*Aucklandia lappa*	- N/A	
*Caesalpinia bonduc*	- N/A	
*Capsicum annuum*	- CYP3A4 and CYP2C9 inhibition - Potent P-glycoprotein inhibition - Increase daunorubicin and vinblastine accumulation in cancer cells and increases anticancer activity of the drugs in KB-C2 cells - Synergistic effects on anticancer activity of 5-FU, cisplatin, docetaxel, erlotinib, and paclitaxel	[19,54,55,56,57,58,59]
*Carum carvi*	- CYP2C9 and CYP3A4 inhibition - UGT1A1 induction	[19,60]
*Cassia fistula*	- N/A	
*Centella asiatica*	- CYP1A2, CYP2B1, CYP2B2, CYP2C19, CYP2C9, CYP2D6, CYP2E1, CYP3A inhibition	[38,61,62,63]
*Cissus quadrangularis*	- N/A	
*Citrus hystrix*	- CYP3A4 and P-glycoprotein inhibition	[64]
*Clerodendrum indicum*	- N/A	
*Clinacanthus nutans*	- N/A	
*Cuminum cyminum*	- CYP2C9 and CYP3A4 inhibition	[19]
*Curcuma longa*	- CYP1A2, CYP2B6, CYP2C9, CYP2C19, CYP2D6, CYP3A, CYP3A4 and P-glycoprotein inhibition	[19,65,66]
*Curcuma* spp.	- N/A	
*Cyanthillium cinereum* (*Vernonia cinerea*)	- CYP1A2, CYP2A6, and CYP2D6 inhibition	[67]
*Dracaena cochinchinensis*	- N/A	
*Eurycoma longifolia*	- CYP2C8 inhibition, weak CYP1A2, CYP2A6, and CYP2C19 inhibition	[68,69]
*Ficus racemosa*	- N/A	
*Foeniculum vulgare*	- CYP2C9, CYP3A4, CYP1A2, CYP2D6 and CYP2E1 inhibition	[19,42,70,71]
*Gynostemma pentaphyllum*	- CYP2D6 (major), CYP2C8, CYP3A4, and CYP2C9 inhibition	[72]
*Harrisonia perforata*	- N/A	
*Hibiscus sabdariffa*	- weak CYP1A2, CYP2C8, CYP2D6, CYP2B6, CYP2E1, CYP2C19, CYP3A4, CYP2C9, and CYP2A6 inhibition	[73]
*Hyptis suaveolens*	- N/A	
*Kaempferia parviflora*	- CYP2D6, CYP1A2, and CYP3A4 inhibition	[74,75]
*Lepidium sativum*	- N/A	
*Ligusticum sinense*	- N/A	
*Mesua ferrea*	- P-glycoprotein inhibition	[76]
*Mimusops elengi*	- N/A	
*Momordica charantia*	- CYP2C9 and P-glycoprotein inhibition	[17,77,78]
*Moringa oleifera*	- CYP1A2 inhibition	[79,80]
*Morus alba*	- CYP3A4, CYP2D6, P-glycoprotein inhibition, and CYP3A4 induction	[52,74,81,82,83]
*Murdannia loriformis*	- N/A	
*Nardostachys jatamansi*	- N/A	
*Nelumbo nucifera*	- CYP1A2, CYP2C19, CYP2C9, CYP2D6, CYP2E1 and CYP3A4 inhibition	[84,85,86]
*Neopicrorhiza scrophulariiflora*	- N/A	
*Nigella sativa*	- CYP1A2, CYP2C9, and CYP3A4, and CYP2C19inhibition (Thymoquinone) - Synergistic effects on anticancer activity of 5-FU, cyclophosphamide, doxorubicin, gemcitabine, and topotecan	[87,88,89,90,91,92,93,94,95]
*Ocimum sanctum*	- N/A	
*Orthosiphon aristatus* (*Orthosiphon stamineus*)	- CYP2C19, CYP2C9, CYP2D6, CYP3A4, UGT1A7, UGT1A1, UGT1A6 and UGT1A8 inhibition - P-glycoprotein inhibition results in decreasing resistance of KB-V-1 cells to vinblastine	[32,38,74,82,96,97]
*Phyllanthus emblica*	- Weak CYP1A2, CYP2C9, CYP2D6, CYP2E1, CYP3A4 inhibition, P-glycoprotein inhibition, and synergistic growth inhibitory effect with cisplatin and doxorubicin	[98,99,100]
*Pimpinella anisum*	- CYP2C9 and CYP3A4 inhibition	[19]
*Piper betle*	- N/A	
*Piper nigrum*	- CYP2C9 and CYP3A4 inhibition - P-glycoprotein, MRP1 and BCRP1 transporter inhibition	[17,19,42,101,102]
*Piper retrofractum*	- N/A	
*Piper sarmentosum*	- N/A	
*Piper wallichii*	- N/A	
*Plantago ovata*	- N/A	
*Pterocarpus santalinus*	- N/A	
*Santalum album*	- CYP3A4 and CYP2D6 inhibition	[42]
*Senna alata*(*Casssia alata*)	- CYP1A2, CYP2C19, CYP2D6, CYP3A4 inhibition	[74,77,103]
*Senna garrettiana*(*Cassia garrettiana)*	- N/A	
*Senna tora*(*Cassia tora*)	- N/A	
*Solanum trilobatum*	- P-glycoprotein inhibition	[99]
*Solori scandens*(*Derris scandens*)	- N/A	
*Tarlmounia elliptica*	- N/A	
*Terminalia bellirica*	- Synergistic effects on growth inhibitory effects of cisplatin in A549 cells and doxorubicin in HepG2 cells	[100]
*Terminalia chebula*	- CYP2E1 and CYP2C19 inhibition	[104]
*Thunbergia laurifolia*	- CYP1A4, CYP2D6 and CYP3A4 inhibition	[74,82,97,105]
*Tiliacora triandra*	- N/A	
*Tinospora crispa*	- CYP3A4 and CYP2D6 inhibition	[42]
*Trachyspermum ammi*	- CYP2C9 and CYP3A4 inhibition	[19]
*Zingiber montanum* (*Zingiber cassumunar*)	- CYP2D6 and CYP3A4 inhibition	[42]
*Zingiber officinale*	- N/A	
*Zingiber zerumbet(Zingiber aromaticum*)	- CYP2D6 and CYP3A4 inhibition	[42]

N/A, Not available.

**Table 2:** Pharmacokinetics-based anticancer-herb interactions with Thai herbs.

Thai Herbs	Effects of Thai Herbal Products	Potential Drug Interaction	Possible Effects on Anticancer Drugs	References
*Aegle marmelos*	CYP1A2 inhibition *In vitro*: Methanolic extract of *Aegle marmelos* inhibits CYP1A2 with IC_50_ = 0.8 μg/mL.	Dasatinib Imatinib	Increase concentrations	[14]
		Dacabarzine Flutamide	Decrease levels of active metabolites	
	CYP3A4 inhibition *In vitro*: Methanolic extract of *Aegle marmelos* inhibits CYP3A4 in pooled human liver microsomes with IC_50_ = 5 μg/mL.	Dasatinib Docetaxel Doxorubicin Etoposide Imatinib Letrozole Megestrol Nilotinib Paclitaxel Vinblastine Vincristine Vinorelbine	Increase concentrations	[14]
		Cyclophosphamide Ifosfamide Tamoxifen	Decrease levels of active metabolites	
*Allium sativum*	CYP1A2 inhibition *In vitro*: Allicin inhibits CYP1A2 with IC_50_ = 44.22 µM.	Dasatinib Imatinib	Increase concentrations	[25]
		Dacabarzine Flutamide	Decrease levels of active metabolites	
	CYP3A4 inhibition *In vitro*: Allicin, apigenin and myricetin inhibit CYP3A4 with IC_50_ = 43.73, 0.4, and 44.5 μM, respectively.	Dasatinib Docetaxel Doxorubicin Etoposide Imatinib Letrozole Megestrol Nilotinib Paclitaxel Vinblastine Vincristine Vinorelbine	Increase concentrations	[20,25]
		Cyclophosphamide Ifosfamide Tamoxifen	Decrease levels of active metabolites	
	CYP2C9 inhibition *In vitro*: Allicin, apigenin, and myricetin inhibit CYP2C9 with IC_50_ = 5.41, 6.4, and 32.1 μM, respectively.	Dasatinib Imatinib	Increase concentrations	[20,25]
		Cyclophosphamide Ifosfamide	Decrease levels of active metabolites	
	CYP2C19 inhibition *In vitro*: Allicin inhibits CYP1A2 with IC_50_ = 3.52 µM.	Imatinib	Increase concentrations	[25]
		Tamoxifen	Decrease levels of active metabolites	
	CYP2D6 inhibition *In vitro*: Allicin inhibits CYP1A2 with IC_50_ = 47.10 µM.	Doxorubicin Imatinib	Increase concentrations	[25]
		Tamoxifen	Decrease levels of active metabolites	
*Andrographis* *paniculata*	CYP1A2 inhibition *In vitro*: Extract of *Andrographis* *paniculata* inhibits CYP1A2 with IC_50_ = 5.1 μg/mL.	Dasatinib Imatinib	Increase concentrations	[39,40]
		Dacabarzine Flutamide	Decrease levels of active metabolites	
	CYP2C19 inhibition *In vitro*: Ethanolic extract of *Andrographis paniculata* inhibits CYP2C19 with IC_50_ = 91.7 μg/mL.	Imatinib	Increase concentrations	[38]
		Tamoxifen	Decrease levels of active metabolites	
	UGT1A1 inhibition *In vitro*: Ethanolic extract of *Andrographis paniculata* inhibits UGT1A1 with IC_50_ = 5.00 µg/mL.	Etoposide Dasatinib	Increase concentrations	[32]
	UGT2B7 inhibition *In vitro*: Spray-dried 50% methanolic powder of *Andrographis paniculata* inhibits UGT2B7 with IC_50_ = 2.82 µg/mL.	Tamoxifen	Decrease levels of active metabolites	[32]
*Anethum graveolens*	CYP3A4 inhibition *In vitro*: 100 µg/mL of *Anethum graveolens* extract inhibit CYP3A4 with percent inhibition more than 50%.	Dasatinib Docetaxel Doxorubicin Etoposide Imatinib Letrozole Megestrol Nilotinib Paclitaxel Vinblastine Vincristine Vinorelbine	Increase concentrations	[19]
		Cyclophosphamide Ifosfamide Tamoxifen	Decrease levels of active metabolites	
*Angelica sinensis*	CYP3A4 induction *In vivo*: Ethanolic crude extract, ligustilide, linoleic acid, ferulic acid, and beta-sitosterol from *Angelica sinensis* induces CYP3A4 activity in HepG2 cells with maximum induction at 118 ± 2.26% relative rifampin.	Dasatinib Docetaxel Doxorubicin Etoposide Imatinib Letrozole Megestrol Nilotinib Paclitaxel Vinblastine Vincristine Vinorelbine	Decrease concentration	[48]
		Cyclophosphamide Ifosfamide Tamoxifen	Increase levels of active metabolites	
*Areca catechu*	CYP3A4 inhibition *In vitro*: 100 μg/mL of *Areca catechu* aqueous extracts inhibits CYP3A4 with percent inhibition 85%	Dasatinib Docetaxel Doxorubicin Etoposide Imatinib Letrozole Megestrol Nilotinib Paclitaxel Vinblastine Vincristine Vinorelbine	Increase concentrations	[49]
		Cyclophosphamide Ifosfamide Tamoxifen	Decrease levels of active metabolites	
*Carum carvi*	CYP2C9 inhibition *In vitro*: 100 μg/mL of *Carum carvi* extract inhibits CYP2C9 with percent inhibition more than 50%.	Dasatinib Imatinib	Increase concentrations	[19]
		Cyclophosphamide Ifosfamide	Decrease levels of active metabolites	
	CYP3A4 inhibition *In vitro*: 100 μg/mL of *Carum carvi* extract inhibits CYP3A4 with percent inhibition more than 50%.	Dasatinib Docetaxel Doxorubicin Etoposide Imatinib Letrozole Megestrol Nilotinib Paclitaxel Vinblastine Vincristine Vinorelbine	Increase concentrations	[19]
		Cyclophosphamide Ifosfamide Tamoxifen	Decrease levels of active metabolites	
*Centella asiatica*	CYP2C19 inhibition *In vitro*: Dichloromethane extract of *Centella asiatica* inhibits CYP2C19 with IC_50_ = 30.2 μg/mL.	Imatinib	Increase concentrations	[38]
		Tamoxifen	Decrease levels of active metabolites	
	CYP2C9 inhibition *In vitro*: Ethanolic extract of *Centella asiatica* inhibits CYP2C9 with IC_50_ = 48.41 ± 4.64 μg/mL.	Dasatinib Imatinib	Increase concentrations	[63]
		Cyclophosphamide Ifosfamide	Decrease levels of active metabolites	
	CYP1A2 inhibition *In vitro*: Ethanolic extract of *Centella asiatica* inhibits CYP1A2 with IC_50_ = 42.23 ± 3.65 μg/mL.	Dasatinib Imatinib	Increase concentrations	[63]
		Dacabarzine Flutamide	Decrease levels of active metabolites	
*Cuminum cyminum*	CYP2C9 inhibition *In vitro*: 100 µg/mL of *Cuminum cyminum* extract inhibits CYP2C9 with percent inhibition more than 50%.	Dasatinib Imatinib	Increase concentrations	[19]
		Cyclophosphamide Ifosfamide	Decrease levels of active metabolites	
	CYP3A4 inhibition *In vitro*: 100 µg/mL of *Cuminum cyminum* extract inhibits CYP3A4 with percent inhibition more than 75%.	Dasatinib Docetaxel Doxorubicin Etoposide Imatinib Letrozole Megestrol Nilotinib Paclitaxel Vinblastine Vincristine Vinorelbine	Increase concentrations	[19]
		Cyclophosphamide Ifosfamide Tamoxifen	Decrease levels of active metabolites	
*Curcuma longa*	CYP1A2 inhibition *In vitro*: Curcumin inhibits CYP1A2 with IC_50_ = 40 µM.	Dasatinib Imatinib	Increase concentrations	[65]
		Dacabarzine Flutamide	Decrease levels of active metabolites	
	CYP2C9 inhibition *In vitro*: Curcumin inhibits CYP2C9 with IC_50_ = 14.8 µg/mL. Aqueous extract of *Curcuma longa* inhibits CYP2C9 with IC_50_ = 82.3 ± 6.05 µg/mL.	Dasatinib Imatinib	Increase concentrations	[19,66]
		Cyclophosphamide Ifosfamide	Decrease levels of active metabolites	
	CYP3A4 inhibition *In vitro*: Extract of *Curcuma longa* inhibits CYP3A4 with IC_50_ = 17 µg/mL. Curcumin inhibits CYP3A4 with IC_50_ = 16.3 µM.	Dasatinib Docetaxel Doxorubicin Etoposide Imatinib Letrozole Megestrol Nilotinib Paclitaxel Vinblastine Vincristine Vinorelbine	Increase concentrations	[19,65]
		Cyclophosphamide Ifosfamide Tamoxifen	Decrease levels of active metabolites	
*Cyanthillium* *cinereum* *(Vernonia cinerea)*	CYP2A6 inhibition *In vitro*: Flavonoid chrysoeriol inhibits CYP2A6 with *K*_i_ = 1.93 ± 0.05 µM, hirsutinolides inhibits CYP2A6 with IC_50_ = 12–23 μM.	Letrozole Tamoxifen	Increase concentrations	[67]
		Ifosfamide	Decrease levels of active metabolites	
	CYP1A2 inhibition *In vitro*: Flavonoid chrysoeriol inhibits CYP1A2 with *K*_i_ = 3.39 ± 0.21 μM.	Dasatinib Imatinib	Increase concentrations	[67]
		Dacarbazine Flutamide	Decrease levels of active metabolites	
	CYP2D6 inhibition *In vitro*: Hirsutinolides inhibits CYP2D6 with IC_50_ = 15–41 μM.	Doxorubicin Imatinib	Increase concentrations	[67]
		Tamoxifen	Decrease levels of active metabolites	
*Foeniculum vulgare*	CYP2C9 inhibition *In vitro*: 100 µg/mL of *Foeniculum vulgare* extract inhibits CYP2C9 with percent inhibition more than 75%.	Dasatinib Imatinib	Increase concentrations	[19]
		Cyclophosphamide Ifosfamide	Decrease levels of active metabolites	
	CYP2D6 inhibition *In vitro*: Water extract of *Foeniculum vulgare* inhibits CYP2D6 with IC_50_ = 23 ± 2 µg/mL.	Doxorubicin Imatinib	Increase concentrations	[70]
		Tamoxifen	Decrease levels of active metabolites	
	CYP2E1 inhibition *In vitro*: Water extract of *Foeniculum vulgare* inhibits CYP2E1 with IC_50_ = 23 ± 4 µg/mL.	Dacarbazine Tamoxifen	Decrease levels of active metabolites	[71]
	CYP3A4 inhibition *In vitro*: 100 µg/mL of *Foeniculum vulgare* extract inhibits CYP3A4 with percent inhibition more than 75%, water extract of *Foeniculum vulgare* inhibits CYP3A4 with IC_50_ = 40 ± 4 µg/mL.	Dasatinib Docetaxel Doxorubicin Etoposide Imatinib Letrozole Megestrol Nilotinib Paclitaxel Vinblastine Vincristine Vinorelbine	Increase concentrations	[19,70]
		Cyclophosphamide Ifosfamide Tamoxifen	Decrease levels of active metabolites	
*Gynostemma* *pentaphyllum*	CYP2D6 inhibition *In vitro*: Gypenosides inhibit CYP2D6 with IC_50_ = 1.61 µg/mL.	Doxorubicin Imatinib	Increase concentrations	[72]
		Tamoxifen	Decrease levels of active metabolites	
	CYP2C8 inhibition *In vitro*: Gypenosides inhibit CYP2C8 with IC_50_ = 20.06 µg/mL.	Nilotinib Paclitaxel Tamoxifen	Increase concentrations	[72]
		Ifosfamide Imatinib	Decrease levels of active metabolites	
	CYP3A4 inhibition *In vitro*: Gypenosides inhibit CYP3A4 with IC_50_ = 34.76 µg/mL.	Dasatinib Docetaxel Doxorubicin Etoposide Imatinib Letrozole Megestrol Nilotinib Paclitaxel Vinblastine Vincristine Vinorelbine	Increase concentrations	[72]
		Cyclophosphamide Ifosfamide Tamoxifen	Decrease levels of active metabolites	
	CYP2C9 inhibition *In vitro*: Gypenosides inhibit CYP2C9 with IC_50_ = 54.52 µg/mL.	Dasatinib Imatinib Tamoxifen	Increase concentrations	[72]
		Cyclophosphamide Ifosfamide	Decrease levels of active metabolites	
*Kaempferia* *parviflora*	CYP1A2 inhibition Patients who used extract from *Kaempferia parviflora* showed CYP1A2 inhibition. It also showed interaction with fluoxetine.	Dasatinib Imatinib	Increase concentrations	[75]
		Dacabarzine Flutamide	Decrease levels of active metabolites	
	CYP2D6 inhibition *In vitro*: Ethanolic extract of *Kaempferia parviflora* inhibits CYP2D6 with IC_50_ = 77 ± 9.54 µg/mL.	Doxorubicin Imatinib	Increase concentrations	[74]
		Tamoxifen	Decrease levels of active metabolites	
	CYP3A4 inhibition *In vitro*: Ethanolic extract of *Kaempferia parviflora* inhibits CYP3A4 with IC_50_ = 28 ± 19.5 µg/mL.	Dasatinib Docetaxel Doxorubicin Etoposide Imatinib Letrozole Megestrol Nilotinib Paclitaxel Vinblastine Vincristine Vinorelbine	Increase concentrations	[74]
		Cyclophosphamide Ifosfamide Tamoxifen	Decrease levels of active metabolites	
*Moringa oleifera*	CYP1A2 inhibition *In vitro*: Ethanolic extract inhibits CYP1A2 with IC_50_ = 13.8 ± 9.8 µg/mL.	Dasatinib Imatinib	Increase concentrations	[80]
		Dacabarzine Flutamide	Decrease levels of active metabolites	
*Nelumbo nucifera*	CYP2C9 inhibition *In vitro*: Alkaloid fraction of *Nelumbo nucifera* inhibits CYP2C9 with IC_50_ = 52.58 µg/mL.	Dasatinib Imatinib	Increase concentrations	[84]
		Cyclophosphamide Ifosfamide	Decrease levels of active metabolites	
	CYP2C19 inhibition *In vitro*: Ethanolic extract of *Nelumbo nucifera* inhibits CYP2C19 with IC_50_ = 77.38 µg/mL. Alkaloid fraction of *Nelumbo nucifera* inhibits CYP2C19 with IC_50_ = 40.79 µg/mL.	Imatinib	Increase concentrations	[84]
		Tamoxifen	Decrease levels of active metabolites	
	CYP2D6 inhibition *In vitro*: Extract of *Nelumbo nucifera* inhibits CYP2D6 with IC_50_ = 12.05 µg/mL^.^ Alkaloid fraction of *Nelumbo nucifera* inhibits CYP2D6 with IC_50_ = 0.96 µg/mL. *In vivo*: Alkaloid fraction of *Nelumbo nucifera* inhibits CYP2D6 in rat.	Doxorubicin Imatinib	Increase concentrations	[84,108]
		Tamoxifen	Decrease levels of active metabolites	
	CYP3A4 inhibition *In vitro*: Extract of *Nelumbo nucifera* inhibits CYP3A4 with IC_50_ = 15.7 ± 2.1 µg/mL.	Dasatinib Docetaxel Doxorubicin Etoposide Imatinib Letrozole Megestrol Nilotinib Paclitaxel Vinblastine Vincristine Vinorelbine	Increase concentrations	[85]
		Cyclophosphamide Ifosfamide Tamoxifen	Decrease levels of active metabolites	
*Nigella sativa*	CYP1A2 inhibition *In vitro*: Thymoquinone inhibits CYP1A2 with IC_50_ 26.5 ± 2.9 µM	Dasatinib Imatinib	Increase concentrations	[88]
		Dacabarzine Flutamide	Decrease levels of active metabolites	
	CYP2C9 inhibition *In vitro*: Thymoquinone inhibits CYP2C9 with IC_50_ 0.5 ± 0.4 µM	Dasatinib Imatinib	Increase concentrations	[88]
		Cyclophosphamide Ifosfamide	Decrease levels of active metabolites	
	CYP3A4 inhibition *In vitro*: Thymoquinone inhibits CYP3A4 with IC_50_ 25.2 ± 3.1 µM	Dasatinib Docetaxel Doxorubicin Etoposide Imatinib Letrozole Megestrol Nilotinib Paclitaxel Vinblastine Vincristine Vinorelbine	Increase concentrations	[88]
		Cyclophosphamide Ifosfamide Tamoxifen	Decrease levels of active metabolites	
	CYP2C19 inhibition *In vitro*: Thymoquinone inhibits CYP2C19 with IC_50_ 3.6 ± 0.9 µM	Imatinib	Increase concentrations	[91]
		Tamoxifen	Decrease levels of active metabolites	
*Orthosiphon aristatus* (*Orthosiphon stamineus*)	CYP2C19 inhibition *In vitro*: Petroleum ether extract of *Orthosiphon aristatus* inhibits CYP2C19 with IC_50_ = 67.1 μg/mL. Sinensetin and eupatorin, active compounds of *Orthosiphon aristatus*, inhibit CYP2C19 with IC_50_ = 71.6 and 12.1 µg/mL, respectively.	Imatinib	Increase concentrations	[38]
		Tamoxifen	Decrease levels of active metabolites	
	CYP2D6 inhibition *In vitro*: Ethanolic extract of *Orthosiphon aristatus* inhibits CYP2D6 with IC_50_ = 31.0 ± 19.5 µg/mL. Eupatorin, an active compound of *Orthosiphon aristatus*, inhibits CYP2D6 with IC_50_ = 3.8 µg/mL.	Doxorubicin Imatinib	Increase concentrations	[74,96]
		Tamoxifen	Decrease levels of active metabolites	
	CYP3A4 inhibition *In vitro*: Dichloromethane and petroleum ether extracts of *Orthosiphon aristatus* inhibit CYP3A4 with IC_50_ = 96.5 and 46.3 µg/mL, respectively. Ethanolic extract of *Orthosiphon aristatus* inhibits CYP3A4 with IC_50_ = 40 ± 8.7 µg/mL. Rosmarinic acid and eupatorin, active compounds of *Orthosiphon aristatus*, inhibit CYP3A4 with IC_50_ = 86.9 and 5.0 µg/mL, respectively.	Dasatinib Docetaxel Doxorubicin Etoposide Imatinib Letrozole Megestrol Nilotinib Paclitaxel Vinblastine Vincristine Vinorelbine	Increase concentrations	[74,96]
		Cyclophosphamide Ifosfamide Tamoxifen	Decrease levels of active metabolites	
	UGT1A1 inhibition *In vitro*: Spray-dried 50% methanolic powder of *Orthosiphon aristatus* inhibits UGT1A1 with IC_50_ = 24.65 µg/mL.	Etoposide Dasatinib	Increase concentrations	[32]
*Pimpinella anisum*	CYP3A4 inhibition *In vitro*: 100 µg/mL of *Pimpinella anisum* extract inhibits CYP3A4 with percent inhibition more than 50%.	Dasatinib Docetaxel Doxorubicin Etoposide Imatinib Letrozole Megestrol Nilotinib Paclitaxel Vinblastine Vincristine Vinorelbine	Increase concentrations	[19]
		Cyclophosphamide Ifosfamide Tamoxifen	Decrease levels of active metabolites	
*Piper nigrum*	CYP2C9 inhibition *In vitro*: Black pepper and white pepper extracts inhibit CYP2C9 with IC_50_ = 12.1 and 3.2 µg/mL, respectively.	Dasatinib Imatinib	Increase concentrations	[19]
		Cyclophosphamide Ifosfamide	Decrease levels of active metabolites	
	CYP3A4 inhibition *In vitro*: Black pepper and white pepper extracts inhibit CYP3A4 with IC_50_ = 4.1 and 1.0 µg/mL, respectively. Methanolic extract from *Piper nigrum* leaves and fruits inhibit CYP3A4 with IC_50_ = 25 and 29 µg/mL, respectively.	Dasatinib Docetaxel Doxorubicin Etoposide Imatinib Letrozole Megestrol Nilotinib Paclitaxel Vinblastine Vincristine Vinorelbine	Increase concentrations	[19,42]
		Cyclophosphamide Ifosfamide Tamoxifen	Decrease levels of active metabolites	
*Senna alata * (*Casssia alata*)	CYP1A2 inhibition *In vitro*: Water extract powder of *Senna alata* inhibits CYP1A2 with IC_50_ = 28.3 ± 2.42 µg/mL.	Dasatinib Imatinib	Increase concentrations	[77]
		Dacabarzine Flutamide	Decrease levels of active metabolites	
	CYP2D6 inhibition *In vitro*: Ethanolic extract of *Senna alata* inhibits CYP2D6 with IC_50_ = 33.0 ± 25.6 µg/mL.	Doxorubicin Imatinib	Increase concentrations	[74,77]
		Cyclophosphamide Ifosfamide Tamoxifen	Decrease levels of active metabolites	
	CYP3A4 inhibition *In vitro*: Ethanolic extract of *Senna alata* inhibits CYP3A4 with IC_50_ = 24.3 ± 14.3 µg/mL.	Dasatinib Docetaxel Doxorubicin Etoposide Imatinib Letrozole Megestrol Nilotinib Paclitaxel Vinblastine Vincristine Vinorelbine	Increase concentrations	[74]
		Cyclophosphamide Ifosfamide Tamoxifen	Decrease levels of active metabolites	
*Trachyspermum ammi*	CYP3A4 inhibition *In vitro*: 100 µg/mL of *Trachyspermum ammi* extract inhibits CYP3A4 with percent inhibition more than 50%.	Dasatinib Docetaxel Doxorubicin Etoposide Imatinib Letrozole Megestrol Nilotinib Paclitaxel Vinblastine Vincristine Vinorelbine	Increase concentrations	[19]
		Cyclophosphamide Ifosfamide Tamoxifen	Decrease levels of active metabolites	
*Thunbergia laurifolia*	CYP2D6 inhibition *In vitro*: Ethanolic extract of *Thunbergia laurifolia* inhibits CYP2D6 with IC_50_ = 45.0 ± 5.0 µg/mL.	Doxorubicin Imatinib	Increase concentrations	[74]
		Cyclophosphamide Ifosfamide Tamoxifen	Decrease levels of active metabolites	
*Zingiber montanum * (*Zingiber cassumunar*)	CYP2D6 inhibition *In vitro*: Extract of *Zingiber montanum* inhibits 25% of CYP2D6 when compare with Quinidine.	Doxorubicin Imatinib	Increase concentrations	[42]
		Tamoxifen	Decrease levels of active metabolites	
	CYP3A4 inhibition *In vitro*: Extract of *Zingiber montanum* inhibits 50% of CYP3A4 when compare with Ketoclonazole.	Dasatinib Docetaxel Doxorubicin Etoposide Imatinib Letrozole Megestrol Nilotinib Paclitaxel Vinblastine Vincristine Vinorelbine	Increase concentrations	[42]
		Cyclophosphamide Ifosfamide Tamoxifen	Decrease levels of active metabolites	

**Table 3:** Lists of anticancer drugs and Thai herbs utilized for the determination of potential DHIs.

Anticancers in 2020 Thailand NLEM	Thai Herbs in 2020 THP
Alkylating drugs Busulfan Chlorambucil Cyclophosphamide Melphalan Carmustine Ifosfamide Procarbazine Cytotoxic antibiotics 8. Bleomycin 9. Dactinomycin 10. Doxorubicin hydrochloride 11. Idarubicin hydrochloride 12. Mitomycin 13. Mitoxantrone hydrochloride Antimetabolites 14. Cytarabine 15. Fluorouracil 16. Mercaptopurine 17. Methotrexate 18. Capecitabine 19. Fludarabine phosphate 20. Gemcitabine hydrochloride 21. Oxaliplatin 22. Tegafur + uracil 23. Tioguanine Vinca alkaloids and etoposide 24. Etoposide 25. Vinblastine 26. Vincristine 27. Vinorelbine Other antineoplastic drugs 28. Asparaginase 29. Cisplatin 30. Carboplatin 31. Hydroxycarbamide 32. Arsenic trioxide 33. Leucovorin calcium 34. Dacarbazine 35. Mitotane 36. Tretinoin 37. Paclitaxel 38. Topotecan 39. Docetaxel 40. Erlotinib 41. Imatinib 42. Nilotinib 43. Dasatinib 44. Rituximab 45. Trastuzumab Sex hormones and hormone antagonists in malignant diseases 46. Tamoxifen 47. Letrozole 48. Megestrol 49. Flutamide 50. Ketoconazole 51. Leuprorelin 52. Triptorelin	1. *Acorus calamus* 2. *Aegle marmelos* 3. *Albizia procera* 4. *Allium ascalonicum* 5. *Allium sativum* 6. *Andrographis paniculata* 7. *Anethum graveolens* 8. *Angelica dahurica* 9. *Angelica sinensis* 10. *Arcangelisia flava* 11. *Areca catechu* 12. *Artemisia annua* 13. *Atractylodes lancea* 14. *Aucklandia lappa* 15. *Caesalpinia bonduc* 16. *Capsicum annuum* 17. *Carum carvi* 18. *Cassia fistula* 19. *Centella asiatica* 20. *Cissus quadrangularis* 21. *Citrus hystrix* 22. *Clerodendrum indicum* 23. *Clinacanthus nutans* 24. *Cuminum cyminum* 25. *Curcuma longa* 26. *Curcuma spp.* 27. *Cyanthillium cinereum* 28. *Dracaena cochinchinensis* 29. *Eurycoma longifolia* 30. *Ficus racemosa* 31. *Foeniculum vulgare* 32. *Gynostemma pentaphyllum* 33. *Harrisonia perforata* 34. *Hibiscus sabdariffa* 35. *Hyptis suaveolens* 36. *Kaempferia parviflora* 37. *Lepidium sativum* 38. *Ligusticum sinense* 39. *Mesua ferrea* 40. *Mimusops elengi* 41. *Momordica charantia* 42. *Moringa oleifera* 43. *Morus alba* 44. *Murdannia loriformis* 45. *Nardostachys jatamansi* 46. *Nelumbo nucifera* 47. *Neopicrorhiza scrophulariiflora* 48. *Nigella sativa* 49. *Ocimum sanctum* 50. *Orthosiphon aristatus* 51. *Phyllanthus emblica* 52. *Pimpinella anisum* 53. *Piper betle* 54. *Piper nigrum* 55. *Piper retrofractum* 56. *Piper sarmentosum* 57. *Piper wallichii* 58. *Plantago ovata* 59. *Pterocarpus santalinus* 60. *Santalum album* 61. *Senna alata* 62. *Senna garrettiana* 63. *Senna tora* 64. *Solanum trilobatum* 65. *Solori scandens* 66. *Tarlmounia elliptica* 67. *Terminalia bellirica* 68. *Terminalia chebula* 69. *Thunbergia laurifolia* 70. *Tiliacora triandra* 71. *Tinospora crispa* 72. *Trachyspermum ammi* 73. *Zingiber montanum* 74. *Zingiber officinale* 75. *Zingiber zerumbet*

## Data Availability

The data has been presented in main text and supplementary material.

## Supplementary Materials

The following are available online at https://www.mdpi.com/article/10.3390/ph15020146/s1. Table S1: Pharmacokinetic profiles of anticancer drugs; Table S2. Definition and classification of the severity level and documentation; Table S3: Thai herbs with anticancer activities.
