# Supplementary material for: Drug-Herb Interactions among Thai Herbs and Anticancer Drugs: A Scoping Review
Source: Pharmaceuticals (Basel). 2022 Jan 26;15(2):146. doi: 10.3390/ph15020146 (PMC8880589; doi:10.3390/ph15020146)
Supplement: Supplementary file 1 [file pharmaceuticals-15-00146-s001.zip › pharmaceuticals-1533299-supplementary.pdf]

**Table S1.** Pharmacokinetic profiles of anticancer drugs.

| Anticancer drugs | Pharmacokinetic profiles | References |
|---|---|---|
| Arsenic trioxide | - Arsenic trioxide undergoes hydrolysis to form arsenious acid, the pharmacologically active species, when placed into solution. Arsenious acid is methylated to less cytotoxic metabolites, monomethylarsonic acid and dimethylarsinic acid, by methyltransferases primarily in the liver. | [1] |
| Asparaginase | - N/A | |
| Bleomycin | - Metabolized by hydrolases, which have been detected in the plasma, liver, spleen, intestine and bone marrow | [2,3] |
| Busulfan | - Primarily metabolized in the liver by glutathione-s-transferases | [4] |
| Capecitabine | - Metabolized by carboxylesterase, cytidine deaminase, and thymidine phosphorylase to fluorouracil | [5-8] |
| Carboplatin | - Excreted primarily by glomerular filtration in urine, with recovery of 65% of a dose within 24 h | [9] |
| Carmustine | - Rapidly degrade both spontaneously and metabolically<br>- Specific metabolites are not identified. | [10,11] |
| Chlorambucil | - Chlorambucil and phenylacetic acid mustard undergo oxidative degradation. | [12] |
| Cisplatin | - The elimination of intact drug and various platinum-containing biotransformation products is via the urine. | [9,13] |
| Cyclophosphamide | - Metabolized by CYP2B6, CYP2C9, and CYP3A4 to yield an active form<br>- Undergoes autoinduction following 2 to 4 days of high-dose intravenous administration of 50 to 60 mg/kg/day | [14] |
| Cytarabine | - Metabolized by deoxycytidine kinase and other nucleotide kinases to aracytidine triphosphate (active); about 86% to 96% of dose is metabolized to inactive uracil arabinoside | [15-17] |
| Dacarbazine | - Extensively metabolized in the liver from prodrug to its active form via CYP1A2 and CYP2E1 | [18-21] |
| Dactinomycin | - Minimally metabolized, approximately 30% of the dose was recovered in urine and feces in one week | [22,23] |
| Dasatinib | - Metabolized in liver via CYP3A4 (major), CYP1A1, CYP1A2, CYP1B1, CYP2C9, CYP3A5, UGT2B15, UGT1A1 and UGT1A9<br>- Efflux transported from hepatocytes via P-glycoprotein and BCRP1 | [24-27] |
| Docetaxel | - Metabolized in liver via CYP3A4 and CYP3A5<br>- Substrate of P-glycoprotein, BCRP1, MRP1, MRP2 and OATP1B3 | [28-32] |

N/A, Not available

**Table S1.** Pharmacokinetic profiles of anticancer drugs (Cont.).

| Anticancer drugs | Pharmacokinetic profiles | References |
|---|---|---|
| Doxorubicin | - Primarily metabolized to doxorubicinol (active), then to inactive aglycones, conjugated sulfates, and glucuronides<br>- Substrate of CYP3A4, CYP2D6, and P-glycoprotein | [33,34] |
| Erlotinib | - Primarily metabolized by CYP3A4 and to a lesser extent by CYP1A2 and CYP1A1 | [35] |
| Etoposide | - Metabolized in liver via CYP3A4, CYP3A5, and UGT1A1 | [36-40] |
| Fludarabine | - Hepatic metabolism via dephosphorylation and phosphorylation by deoxycytidine kinase to active metabolite: 2-fluoro-9-beta-D-arabinofuranosyladenine | [41,42] |
| Fluorouracil | - Metabolized by dihydropyrimidine dehydrogenase | [43,44] |
| Flutamide | - Metabolized in liver via CYP1A2 from prodrug to its active form | [45-47] |
| Gemcitabine | - Metabolized intracellularly by nucleoside kinases to the active diphosphate and triphosphate nucleosides | [48,49] |
| Hydroxycarbamide (Hydroxyurea) | - Oral absorption of the drug is virtually complete, the volume of distribution is equivalent to total body water and elimination is through both renal and nonrenal mechanisms. | [50-52] |
| Idarubicin | - Extensively metabolized to an active metabolite, idarubicinol, which is slowly eliminated with a plasma T½ ranging between 41 – 69 hours. The drug is eliminated by biliary and renal excretion, mostly in the form of idarubicinol.<br>- Substrate of P-glycoprotein | [53-55] |
| Ifosfamide | - Metabolized by CYP3A4 (main) and 2B6 (main) with 2A6, 2C8, 2C9 and 2C19 to be active forms<br>- Autoinduction by transcriptional upregulation of CYP3A4 | [56-59] |
| Imatinib | - Efflux transported from cancer cells via P-glycoprotein and BCRP1<br>- Metabolized in liver via CYP3A4 (major), CYP3A5 (major), CYP2C8, CYP1A1 and CYP4F2 to active metabolite with similar potency to its parent compound<br>- Metabolized in liver via CYP1A2, CYP1B1, CYP2C9, CYP2C19 and CYP2D6 to inactive metabolites | [27,60,61] |
| Ketaconazole | - Metabolized by CYP3A4, oxidative *O*-dealkylation, and aromatic hydroxylation<br>- Potent inhibitor and substrate of CYP3A4 | [62] |
| Letrozole | - Metabolized in liver via CYP3A4 and CYP2A6 | [63-65] |
| Leucovorin calcium | - Leucovorin (a mixture of d- and l-diasteroisomers), only the l-isomer is metabolized to intermediate active folates and N-5-methyltetrahydrofolate and the d-isomer is not metabolized and lacks intrinsic biological activity.<br>- The inactive isomer d-formyltetrahydrofolate is largely eliminated unchanged in the urine | [66,67] |

**Table S1.** Pharmacokinetic profiles of anticancer drugs (Cont.).

| Anticancer drugs | Pharmacokinetic profiles | References |
|---|---|---|
| Leucovorin calcium (Cont.) | while the active isomer l-formyltetrahydrofolate undergoes both urinary excretion and extensive metabolism. | [66] |
| Leuprorelin | - N/A | |
| Megestrol | - Metabolized in liver via CYP3A4 and UGT2B17 | [68-70] |
| Melphalan | - Chemical hydrolysis to monohydroxymelphal (inactive form), one of the primary metabolites of melphalan | [71-73] |
| Mercaptopurine | - Activated via hypoxanthine-guanine phosphoribosyl transferase and several enzymes to form 6-thioguanine nucleotides, which is responsible for efficacy of mercaptopurine in cell-cycle arrest and cell death. It also undergoes thiol methylation catalyzed by polymorphic enzyme thiopurine S-methyltransferase and oxidation by xanthine oxidase to form the inactive metabolite, methylmercaptopurine and 6-thiouric acid respectively. | [74-77] |
| Methotrexate | - Excretion takes place mainly via the kidneys. Approximately 41% of the dose is excreted unchanged in the urine within the first six hours, 90% within 24 hours. | [78-81] |
| Mitomycin | - The main location for metabolism and elimination is the liver. Accordingly, high concentrations of mitomycin have been found in the gall bladder. | [82,83] |
| Mitotane | - After intravenous administration, 25% of the dose was excreted as metabolites within 24 hours. Following discontinuation of mitotane treatment, it is slowly released from storage sites in fat, leading to reported terminal plasma half-lives ranging from 18 to 159 days. | [84,85] |
| Mitoxantrone | - The pathways leading to the metabolism of mitoxantrone have not been elucidated. Mitoxantrone is excreted slowly in urine and feces as either unchanged active substance or as inactive metabolites.<br>- Substrate of BCRP | [86-88] |
| Nilotinib | - Metabolized in liver via CYP3A4 (major) and CYP2C8<br>- Efflux transported from hepatocytes via P-glycoprotein and BCRP1 | [27,89-91] |
| Oxaliplatin | - Platinum is predominantly excreted in urine, with clearance mainly in the 48 hours following administration. By day 5, approximately 54% of the total dose was recovered in the urine and < 3% in the feces. | [9] |
| Paclitaxel | - Appears to be metabolized primarily by cytochrome P450 enzymes. Following administration of a radio-labelled paclitaxel, an average of 26, 2 and 6% of the radioactivity was excreted in the feces as 6$\alpha$-hydroxy-paclitaxel, 3'-p-hydroxypaclitaxel, and 6$\alpha$-3'-p-dihydroxy-paclitaxel, respectively. The formation of these hydroxylated metabolites is catalyzed by CYP2C8, CYP3A4, and both CYP2C8 and CYP3A4, respectively.<br>- Substrate of P-glycoprotein, BCRP1, MRP1 and MRP2<br>- Transport into hepatocytes via OATP1B3 | [31,92,93] |

N/A, Not available

**Table S1.** Pharmacokinetic profiles of anticancer drugs (Cont.).

| Anticancer drugs | Pharmacokinetic profiles | References |
|---|---|---|
| Procarbazine | - Rapidly metabolized, the primary circulating metabolite is the azo derivative while the major urinary metabolite has been shown to be N-isopropyl-terephthalamic acid. | [94-97] |
| Rituximab | - N/A | |
| Tamoxifen | - Metabolized in liver via CYP3A4, CYP3A5, CYP2D6, CYP2C8, CYP2C9, CYP2C19, CYP2B6, CYP2A6, CYP2E1, SULT1A1, UGT2B7 and UGT1A4<br>- 92% of tamoxifen are metabolized via CYP3A4 and CYP3A5 to N-desmethyl tamoxifen, an active metabolite with potency similar to parent compound.<br>- 6% of tamoxifen are metabolized via CYP2D6 to 4-Hydroxytamoxifen, an active metabolite with 30 -100 times higher potency than the parent compound.<br>- Efflux transported from hepatocytes via P-glycoprotein and BCRP1 | [98-103] |
| Tegafur + uracil | - N/A | |
| Tioguanine | - Hepatic metabolism: rapidly and extensively metabolized via thiopurine methyltransferase to 2-amino-6-methylthioguanine (active) and inactive compounds. | [104,105] |
| Topotecan | - Metabolized in liver by demethylation and in plasma by hydrolysis<br>- Substrate of P- glycoprotein and BCRP | [106,107] |
| Trastuzumab | - N/A | |
| Tretinoin | - Metabolized via CYP450s | [108,109] |
| Triptorelin | - N/A | |
| Vinblastine | - Metabolized in liver via CYP3A subfamily mainly CYP3A4 | [110] |
| Vincristine | - Metabolized in liver via CYP3A subfamily mainly CYP3A4 | [111] |
| Vinorelbine | - Metabolized in liver via CYP3A subfamily mainly CYP3A4 | [112] |

N/A, Not available

**Table S2.** Definition and classification of the severity level and documentation.

| Classification | Definition |
|---|---|
| Severity: Contraindicated | The drug and herb are contraindicated for concurrent use. |
| Severity: Major | The interaction may be life-threatening and/or require medical intervention to minimize or prevent serious adverse effects. |
| Severity: Moderate | The interaction may result in exacerbation of the patient's condition and/or require an alteration in therapy. |
| Severity: Minor | The interactions have limited clinical effects. Manifestations may include an increase in the frequency or severity of the side effects. |
| Severity: Unknown | There is no evidence or report on the interaction, pharmacologic considerations lead clinicians to suspect the interaction exists or drug-herb interaction is unknown. |
| Documentation: Excellent | Controlled studies have clearly established the existence of the interaction. |
| Documentation: Good | Documentation strongly suggests the interaction exists, but well-controlled studies are lacking. |
| Documentation: Fair | Available documentation is poor, but pharmacologic considerations lead clinicians to suspect the interaction exists. |
| Documentation: Unknown | No documentation or no suspected interaction exists. |

**Table S3.** Thai herbs with anticancer activities.

| Thai herbs | Fractions or compounds | Cancer cell types | Activities | References |
|---|---|---|---|---|
| *Acorus calamus* | Acetone and ethyl acetate extract | HT-29 human colorectal cancer cells | - Antiproliferative | [113] |
| | Aqueous extract | HT-29 human colorectal cancer cells | - Increase proliferation | [113] |
| | β-asarone | HT29 and SW480 human colorectal cancer cells | - Induce cell senescence by upregulating lamin B1 expression | [114] |
| *Albizia procera* | 3-O-[β-D-xylopyranosyl-(1→2)-α-L-arabinopyranosyl-(1→6)-2-acetamido-2-deoxy-β-D-glucopyranosyl] echinocystic acid | HepG2 human liver carcinoma cells | - Reduce cell viability | [115] |
| | 3-O-[α-L-arabinopyranosyl-(1→2)-α-L-arabinopyranosyl-(1→6)-2-acetamido-2-deoxy-β-D-glucopyranosyl] echinocystic acid | HepG2 human liver carcinoma cells | - Reduce cell viability | [115] |
| *Allium sativum* | Diallyl trisulfide | A549 human lung adenocarcinoma cells<br>*in vivo*<br>A549 human lung adenocarcinoma cells xenografts in nude mice | - Induce apoptosis and inhibit cell proliferation | [116] |
| *Andrographis paniculata* | Andrographolide | CL1-5 and H358<br>*in vivo*<br>A549 human lung cancer cells xenografts in SCID mice | - Suppress cell proliferation and induce apoptosis and cell cycle arrest<br>- Decrease cancer cell invasion and MMP2 expression<br>- Reduce tumor growth | [117] |
| | Ethanolic extract and andrographolide | B16-F10 mice skin melanoma cells<br>*in vivo*<br>C57BL/6 mice | - Reduce tumor directed capillaries by decreasing level of VEGF | [118] |
| *Angelica dahurica* | Imperatorin | H23, H292 and A549 human lung cancer cells | - Sensitize anoikis by upregulating p53 expression | [119] |
| | | HepG2 human hepatocarcinoma cells<br>*in vivo*<br>HepG2 human hepatocarcinoma cells xenografts in nude mice | - Induce apoptosis and decrease tumor growth | [120] |

**Table S3.** Thai herbs with anticancer activities (Cont.).

| Thai herbs | Fractions or compounds | Cancer cell types | Activities | References |
|---|---|---|---|---|
| *Angelica sinensis* | n-butylidenephtalide | DBTRG-05G human glioblastoma cells<br>*in vivo*<br>DBTRG-05G human glioblastoma cells xenografts in nude mice | - Induce apoptosis and G0/G1 phase cell cycle arrest by upregulating p21 and p27 | [121] |
| | Z-ligustilide | L1210 mouse lymphocyt-ichuman leukemia and K562 leukemia cells | - Induce cell death | [122] |
| *Atractylodes lancea* | Ethanolic extract | CL-6 human cholangiocarinoma, Hep-2 human laryngeal carcinoma, and HepG2 human hepatocarcinoma cells | - Reduce cell viability | [123] |
| | Ethanolic extract containing 6.64% *R*-eudesmol | *in vivo*<br>CL-6 human cholangiocarinoma xenografts in nude mice | - Decrease tumor size | [124] |
| *Capsicum annuum* | Methanolic extract of fruits | HT-29 human colon carcinoma cells | - Decrease cell viability and increase LDH release | [125] |
| | Capsaicin | W480, LoVo and HCT-116 human colorectal cells | - Decrease cell proliferation by suppressing transcriptional activity of *R*-catenin | [126] |
| | | HL60 human leukemia cells | - Decrease cell growth and induce DNA fragmentation | [127] |
| | | U-87 MG human glioblastoma cells | - Induce apoptosis by activating p-38 MAPK pathway | [128] |
| | | KB human cancer cells | - Induce apoptosis and cell cycle arrest | [129] |
| *Carum carvi* | Ethanolic extract of powder fruits | ML-1, J-45.01, EOL, HL-60, 1301, H-9, U266, WICL, C-8166 human leukemia cells | - Decrease cell viability except for HL-60 and U266130 | [130] |
| | Given as diet | *in vivo*<br>Colon cancer in Wistar rats | - Reduce tumor incidence and inhibit tumorigenesis | [131,132] |
| | Carvone | N2a rat neuroblastoma cells | - Reduce cell viability | [133] |
| *Centella asiatica* | Methanolic extract | MCF-7 human breast cancer cells | - Reduce cell viability and induce apoptosis and DNA strand break | [134] |
| | Asiatic acid | SW480 human colon cancer cells | - Induce apoptosis and cell cycle arrest | [135] |

**Table S3.** Thai herbs with anticancer activities (Cont.).

| Thai herbs | Fractions or compounds | Cancer cell types | Activities | References |
|---|---|---|---|---|
| *Clerodendrum indicum* | Oleanolic acid 3-acetate | SW620 human colorectal adenocarcinoma cells, ChaGo-K1 human lung bronchus carcinoma cells, HepG2 human liver carcinoma cells, KATO-III human gastric carcinoma cells, BT-474 human breast carcinoma cells | - Reduce cell viability | [136] |
| *Cuminum cyminum* | Seeds given as diet | *in vivo*<br>Chemical induced forestomach and uterine cervix tumors in mice | - Decrease tumor burden and inhibit tumor multiplication | [137] |
| *Curcuma longa* | Curcumin | A549 and H1299 human lung cancer cells | - Increase apoptosis | [138] |
| | | RS4;11 and REH human leukemia cells | - Increase apoptosis and cell cycle arrest | [139] |
| | Curcumin, Tumerone | HepG2 human hepatocarcinoma cells, MCF-7 human breast cancer cells | -Reduce cell viability | [140] |
| | | MDA-MB-231 human breast cancer cells | - Reduce cell viability and induce apoptosis and cell cycle arrest | [140] |
| *Cyanthillium cinereum* (*Vernonia cinerea*) | Dichloromethane enriched fraction | HeLa, A549, MCF-7, and Caco-2 human epithelial cancer cells | - Reduce cell viability<br>- Increase early and late apoptosis,<br>- Inhibit MDR transporters (ABC-B1 and ABC-G2)<br>- Enhance daunorubicin-uptake in cancer cells | [141] |
| | Methanol and dichloromethane extract, 8$\alpha$-tigloyloxy-hirsutinolide-13-*O*-acetate | HT29 human colon adenocarcinoma cell and HepG2 human liver carcinoma cells | - Reduce cell viability | [142] |
| | Vernolide-A | KB human oral cancer cells, DLD-1 human colon adenocarcinoma cells, NCI-661 human lung cancer cells, and HeLa cells | - Reduce cell viability | [143] |
| | | B16F-10 melanoma cells<br>*in vivo*<br>B16F-10 melanoma cells in C57BL/6 mice | - Reduce cell viability<br>- Downregulate the expression of MMP-2, MMP-9, ERK-1, ERK-2, and VEGF<br>- Inhibit lung metastasis | [144] |

**Table S3.** Thai herbs with anticancer activities (Cont.).

| Thai herbs | Fractions or compounds | Cancer cell types | Activities | References |
|---|---|---|---|---|
| *Cyanthillium cinereum* (*Vernonia cinerea*) (Cont.) | Vernolide-B | KB human oral cancer cells, DLD-1 human colon adenocarcinoma cells, NCI-661 human lung cancer cells, and HeLa cells | - Reduce cell viability | [143] |
| | Methanolic extract and Vernolide-A | EL4 mice T lymphoblast cells and BALB/c mice | - Enhance natural killer cell activity, ADCC, ACC, cytotoxic, and T lymphocyte production in both normal and tumor-bearing animals<br>- enhanced secretion of IL-2 and IFN-γ | [145] |
| *Eurycoma longifolia* | Methanolic extract | HT-1080 human fibrosarcoma, HeLa human cervical cancer, A549 human lung cancer, 26-L5 murine colon cancer, LLC murine lung cancer cells | - Reduce cell viability | [146] |
| | Eurycomanone | HepG2 human hepatocarcinoma cells and HeLa human cervical cancer cells | - Induce apoptosis | [147,148] |
| *Ficus racemosa* | Ethanolic extract | 1BR3 human skin fibroblasts cells, HepG2 human liver carcinoma cells, and HL-60 human leukemia cells | - Reduce cell viability | [149] |
| | Acetone extract | *in vivo*<br>Wistar rat | - Protect renal and testicular from doxorubicin<br>exposure | [150] |
| *Foeniculum vulgare* | Ethanolic extract of powder fruits | L-1, J-45.01, EOL, HL-60, 1301, H-9, U266, WICL, C-8166 human leukemia cells | - Decrease cell viability except for HL-60 and U266 | [130] |
| | Ground seeds | *in vivo*<br>Swiss albino mice | - Decrease incidence and multiplicity of skin and forestomach tumors | [151] |
| | Anethole | HT-1080 human fibrosarcoma cells | - Reduce cell viability, migration and invasion | [152] |
| *Gynostemma pentaphyllum* | Saponin and flavonoid fraction | PC-3 human prostate cancer cells | - Reduce cell viability, induce cell cycle arrest of S and G2/M phases, apoptosis through mitochondria pathway | [153] |

**Table S3.** Thai herbs with anticancer activities (Cont.).

| Thai herbs | Fractions or compounds | Cancer cell types | Activities | References |
|---|---|---|---|---|
| *Gynostemma pentaphyllum* (Cont.) | Gypenosides | *In vitro*, *in vivo*, and clinical use<br>Breast, lung, and multiple type cancers | - Anti-cancer activity | [154] |
| | | *in vitro* and *in vivo* in human myeloid leukemia HL-60 cells, human tongue cancer SCC-4 cells | - Reduce cell viability<br>- Induces cell cycle arrest and apoptosis | [155,156] |
| | Gypenoside L | human hepatocellular carcinoma cells | - Induce ROS–ER–Ca2+–mediated cytoplasmic vacuolation death in cell | [157] |
| *Harrisonia perforata* | Harrisotones A–C, and harrisonol A | P-388 mouse leukemia cells and/or A549 human lung cancer cells | - Reduce cell viability | [158] |
| *Mimusops elengi* | Ethanolic extract | CL-6 human cholangio-carinoma, Hep-2 human laryngeal carcinoma, and HepG2 human hepatocarcinoma cells | - Reduce cell viability | [123] |
| *Momordica charantia* | Crude extract | PC-3 and LNCap human prostate cancer cells and TRAMP mice | - Induce apoptosis and cell cycle arrest<br>- Decrease prostate cancer progression | [159] |
| *Moringa oleifera* | Aqueous extract | KB human oral cancer cells | - Reduce cell viability<br>- Induce apoptosis and DNA fragmentation | [160] |
| *Murdannia loriformis* | Ethanolic extract | MCF-7 human breast cancer cells and HT-29 human colorectal cancer cells | - Reduce cell viability | [161] |
| | 1-β-*O*-D-glucopyranosyl-2-(2'-hydroxy-6'-ene-cosamide)-sphingosine | BT474 human breast cancer cells and<br>SW620 human colorectal adenocarcinoma cells | - Reduce cell viability | [162] |
| *Nardostachys jatamansi* | Methanolic extract | MCF-7 and MDA-MB-231 human breast cancer cells | - Induce apoptosis and cell cycle arrest | [163] |
| *Nelumbo nucifera* | Aqueous extract of leaves | MDA-MB-231 human breast cancer cells | - Reduce cell migration, invasion, MMP2 activity and VEGF expression | [164] |
| | Neferine | H520, H661, H441, and A549 human lung cancer cells | - Reduce cell viability and induce apoptosis | [165] |
| *Nigella sativa* | Thymoquinone | HCT-116 human colon, HeLa human cervical and U251 human glioblastoma cells | - Decrease cell viability | [166] |

**Table S3.** Thai herbs with anticancer activities (Cont.).

| Thai herbs | Fractions or compounds | Cancer cell types | Activities | References |
|---|---|---|---|---|
| *Nigella sativa* (Cont.) | Thymoquinone | HepG2 human hepato-carcinoma cells | - Decrease cell viability<br>- Increase apoptosis and induce cell cycle arrest | [166] |
| | | HuCCT1 human cholangi-ocarcinoma cells<br>*in vivo*<br>HuCCT1 human cholangi-ocarcinoma cells xenografts in mice | - Reduce cell viability and induce cell cycle arrest<br>- Reduce tumor size | [167] |
| | | SASVO3 head and neck squamous cell carcinoma<br>*in vivo*<br>SASVO3 head and neck squamous cell carcinoma xenografts in mice | - Induce apoptosis and cell cycle arrest<br>- Reduce tumor size | [168] |
| | | HT-29 human colon cancer cells | - Increase apoptotic effects of topotecan | [169] |
| *Orthosiphon aristatus* (*Orthosiphon stamineus*) | Methanolic extract | HT-29 human colon and MCF-7 human breast cancer cells | - Reduce cell viability | [170] |
| | Eupatorin | RPMI8226 multiple mye-loma, HL60, MOLT-4 and K562 leukemia and MCF-7 human breast cancer cells | - Reduce cell viability | [171] |
| | | HeLa human cervical cancer cells | - Reduce cell viability<br>- Induce apoptosis and cell cycle arrest | [171] |
| *Phyllanthus emblica* | Phenolic compounds from methanolic extract | MCF-7 human breast cancer cells | - Reduce cell viability | [172] |
| *Piper betle* | Hydroxychavicol | MCF-7 human breast can-cer, HepG2 human hepato-carcinoma, MIA PaCa-2 human pancreatic cancer, and SK-N-SH neuroblas-toma cells | - Reduce cell viability | [173] |
| | | HL-60 human leukemia cells | - Reduce cell viability<br>- Induce cell cycle arrest and apoptotic bodies | [173] |
| | | PC-3, C4-2, and 22Rv1 human prostate cancer cells | - Reduce cell viability<br>- Induce cell cycle arrest and apoptotic bodies | [174] |
| *Piper nigrum* | Ethanolic extract of fruits | MCF-7 human breast and HT-29 human colon cancer cells | - Decrease cell prolifera-tion | [175] |
| | | Ehrlich ascites carcinoma in Balb/c mice | - Increase survival rate | [175] |

**Table S3.** Thai herbs with anticancer activities (Cont.).

| Thai herbs | Fractions or compounds | Cancer cell types | Activities | References |
|---|---|---|---|---|
| *Piper nigrum* (Cont.) | Piperine | HepG2 human hepatocarcinoma cells | - Induce apoptosis | [176] |
| | | Diethylnitrosamine-induced hepatocellular carcinoma in Wistar rats | - Protective effects | [176] |
| | | SKBR3 and BT-474 human breast cancer cells | - Reduce cell viability and MMP9 expression | [177] |
| *Piper sarmentosum* | Ethanolic extract | HepG2 human hepatocarcinoma cells | - Reduce cell viability and induce apoptosis | [178] |
| *Plantago ovata* | Fermented *P. ovata* | HCT-116, LoVo, SW480, HT-29, and Caco2 human colon cancer cells | - Induce apoptosis | [179] |
| *Senna alata* (*Cassia alata*) | Hexane extract | MCF-7 human breast, T24 human bladder and Col 2 colon cancer cells | - Reduce cell viability and induce apoptosis | [180] |
| *Senna garrettiana* (*Cassia garrettiana*) | Cassialoin | HUVEC and colon 26-bearing mice | - Inhibit tumor growth and metastasis | [181] |
| *Solori scandens* (*Derris scandens*) | Ethyl acetate extract and isoflavones | KB human oral cancer, MCF-7 human breast cancer, and NCI-H187 human lung cancer cells | - Reduce cell viability | [182] |
| *Terminalia bellirica* | Ethyl acetate fraction of fruits | HSC-T6 rat hepatic stellate cells | - Reduce cell viability and induce apoptosis | [183] |
| *Terminalia chebula* | Aqueous extract | Jurkat human lymphoblastic T cells | - Reduce cell viability | [184] |
| | Chebulagic acid | Y79 human retinoblastoma cells | - Reduce cell viability, and induce apoptosis and cell cycle arrest | [185] |
| *Tinospora crispa* | Methanolic extract | HT-1080 human fibrosarcoma cells | - Reduce cell viability | [146] |
| *Zingiber officinale* Roscoe | Ethanolic extract | CL-6 human cholangiocarinoma, HepG2 human hepatocarcinoma, and Hep-2 human laryngeal carcinoma, cells | - Reduce cell viability | [123] |
| | Ethanolic extract containing 6.18% of 6-gingerol | CL-6 human cholangiocarinoma xenografts in nude mice | - Decrease tumor size | [124] |
| | 6-gingerol | KB and SCC4 human oral squamous carcinoma and HeLa human cervical cancer cells | - Increase apoptosis and induce cell cycle arrest<br>- Additive cytotoxic effect with cisplatin and wortmannin, phosphoinositide 3-kinase inhibitor | [186] |

## References


1. Product Information: TRISENOX(R) IV injection, arsenic trioxide IV injection. **2010**.
2. Fujita, H. Comparative studies on the blood level, tissue distribution, excretion and inactivation of anticancer drugs. *Jpn J Clin Oncol* **1971**, *1*, 151-162, doi:10.1093/oxfordjournals.jjco.a039354.
3. Umezawa, H. Chemistry and mechanism of action of bleomycin. *Fed Proc* **1974**, *33*, 2296-2302.
4. Product Information: MYLERLAN, busulfan (tablets). **2003**.
5. Product Information: XELODA oral tablets, capecitabine oral tablets. **2009**.
6. Bajetta, E.; Carnaghi, C.; Somma, L.; Stampino, C.G. A pilot safety study of capecitabine, a new oral fluoropyrimidine, in patients with advanced neoplastic disease. *Tumori* **1996**, *82*, 450-452.
7. Rustum, Y.M.; Harstrick, A.; Cao, S.; Vanhoefer, U.; Yin, M.B.; Wilke, H.; Seeber, S. Thymidylate synthase inhibitors in cancer therapy: direct and indirect inhibitors. *J Clin Oncol* **1997**, *15*, 389-400, doi:10.1200/JCO.1997.15.1.389.
8. Thorn, C.F.; Marsh, S.; Carrillo, M.W.; McLeod, H.L.; Klein, T.E.; Altman, R.B. PharmGKB summary: fluoropyrimidine pathways. *Pharmacogenet Genomics* **2011**, *21*, 237-242, doi:10.1097/FPC.0b013e32833c6107.
9. Marsh, S.; McLeod, H.; Dolan, E.; Shukla, S.J.; Rabik, C.A.; Gong, L.; Hernandez-Boussard, T.; Lou, X.J.; Klein, T.E.; Altman, R.B. Platinum pathway. *Pharmacogenet Genomics* **2009**, *19*, 563-564, doi:10.1097/FPC.0b013e32832e0ed7.
10. Product Information: GLIADEL WAFER implant wafer, polifeprosan 20 with carmustine implant wafer. **2006**.
11. Product Information: BICNU IV injection, carmustine IV injection. **2007**.
12. Product Information: LEUKERAN oral tablets, chlorambucil oral tablets. **2011**.
13. Balis, F.M.; Holcenberg, J.S.; Bleyer, W.A. Clinical pharmacokinetics of commonly used anticancer drugs. *Clin Pharmacokinet* **1983**, *8*, 202-232, doi:10.2165/00003088-198308030-00002.
14. Boddy, A.V.; Yule, S.M. Metabolism and pharmacokinetics of oxazaphosphorines. *Clin Pharmacokinet* **2000**, *38*, 291-304, doi:10.2165/00003088-200038040-00001.
15. Creasey, W.A.; Papac, R.J.; Markiw, M.E.; Calabresi, P.; Welch, A.D. Biochemical and pharmacological studies with 1-β-D-arabinofuranosylcytosine in man. *Biochem Pharmacol* **1966**, *15*, 1417-1428, doi:10.1016/0006-2952(66)90186-9.
16. Gilman, A.G.; Rall, T.W.; Nies, A.S.; Taylor, P. *Goodman and Gilman's The Pharmacological Basis of Therapeutics*, 8th ed.; Pergamon: New York, 1990.
17. Product Information: CYTOSAR-U, cytarabine. **1999**.
18. Product Information: DACARBAZINE IV injection, dacarbazine IV injection. **2007**.
19. BC Cancer Agency Cancer Drug Manual. Dacarbazine. Available online: http://www.bccancer.bc.ca/drug-database-site/Drug%20Index/Dacarbazine_monograph_1June2013_formatted.pdf (accessed on 16 August 2020).
20. Housholder, G.E.; Loo, T.L. Disposition of 5-(3,3-dimethyl-1-triazeno)imidazole-4-carboxamide, a new antitumor agent. *J Pharmacol Exp Ther* **1971**, *179*, 386-395.
21. Kantrowitz-Gordon, I.; Hays, K.; Kayode, O.; Kumar, A.R.; Kaplan, H.G.; Reid, J.M.; Safgren, S.L.; Ames, M.M.; Easterling, T.R.; Hebert, M.F. Pharmacokinetics of dacarbazine (DTIC) in pregnancy. *Cancer Chemother Pharmacol* **2018**, *81*, 455-460, doi:10.1007/s00280-017-3511-6.
22. Product Information: Cosmegen, dactinomycin injection. **2004**.
23. Tattersall, M.H.; Sodergren, J.E.; Dengupta, S.K.; Trites, D.H.; Modest, E.J.; Frei, E., 3rd. Pharmacokinetics of actinomycin D in patients with malignant melanoma. *Clin Pharmacol Ther* **1975**, *17*, 701-708, doi:10.1002/cpt1975176701.
24. Product Information: SPRYCEL oral tablets, dasatinib oral tablets. **2008**.
25. Product Information: SPRYCEL oral tablets, dasatinib oral tablets. **2017**.
26. BC Cancer Agency Cancer Drug Manual. Dasatinib. Available online: http://www.bccancer.bc.ca/drug-database-site/Drug%20Index/Dasatinib_monograph_1Mar2017.pdf (accessed on 16 August 2020).
27. Whirl-Carrillo, M.; McDonagh, E.M.; Hebert, J.M.; Gong, L.; Sangkuhl, K.; Thorn, C.F.; Altman, R.B.; Klein, T.E. Pharmacogenomics knowledge for personalized medicine. *Clin Pharmacol Ther* **2012**, *92*, 414-417, doi:10.1038/clpt.2012.96.
28. BC Cancer Agency Cancer Drug Manual. Docetaxel. Available online: http://www.bccancer.bc.ca/drug-database-site/Drug%20Index/Docetaxel_monograph.pdf (accessed on 16 August 2021).
29. Bissett, D.; Kaye, S.B. Taxol and taxotere--current status and future prospects. *Eur J Cancer* **1993**, *29A*, 1228-1231, doi:10.1016/0959-8049(93)90062-k.
30. Hirth, J.; Watkins, P.B.; Strawderman, M.; Schott, A.; Bruno, R.; Baker, L.H. The effect of an individual's cytochrome CYP3A4 activity on docetaxel clearance. *Clin Cancer Res* **2000**, *6*, 1255-1258.
31. Oshiro, C.; Marsh, S.; McLeod, H.; Carrillo, M.W.; Klein, T.; Altman, R. Taxane pathway. *Pharmacogenet Genomics* **2009**, *19*, 979-983, doi:10.1097/FPC.0b013e3283335277.
32. Pazdur, R.; Newman, R.A.; Newman, B.M.; Fuentes, A.; Benvenuto, J.; Bready, B.; Moore, D., Jr.; Jaiyesimi, I.; Vreeland, F.; Bayssas, M.M.; et al. Phase I trial of Taxotere: five-day schedule. *J Natl Cancer Inst* **1992**, *84*, 1781-1788, doi:10.1093/jnci/84.23.1781.
33. Product Information: doxorubicin HCl intravenous injection, doxorubicin HCl intravenous injection. **2013**.
34. Product Information: doxorubicin hydrochloride IV injection, doxorubicin hydrochloride IV injection. **2010**.
35. Product Information: TARCEVA(R) oral tablets, erlotinib oral tablets. **2013**.
36. Creaven, P.J. The clinical pharmacology of VM26 and VP16-213. A brief overview. *Cancer Chemother Pharmacol* **1982**, *7*, 133-140, doi:10.1007/BF00254535.
37. Evans, W.E.; Sinkule, J.A.; Crom, W.R.; Dow, L.; Look, A.T.; Rivera, G. Pharmacokinetics of teniposide (VM26) and etoposide (VP16-213) in children with cancer. *Cancer Chemother Pharmacol* **1982**, *7*, 147-150, doi:10.1007/BF00254537.

38. Pelsor, F.R.; Allen, L.M.; Creaven, P.J. Multicompartment pharmacokinetic model of 4'-demethylepipodophyllotoxin 9-(4,6-*O*-ethylidene-β-D-glucopyranoside) in humans. *J Pharm Sci* **1978**, *67*, 1106-1108, doi:10.1002/jps.2600670821.
39. Rozencweig, M.; Von Hoff, D.D.; Henney, J.E.; Muggia, F.M. VM 26 and VP 16-213: a comparative analysis. *Cancer* **1977**, *40*, 334-342, doi:10.1002/1097-0142(197707)40:1<334::aid-cncr2820400147>3.0.co;2-x.
40. Yang, J.; Bogni, A.; Schuetz, E.G.; Ratain, M.; Dolan, M.E.; McLeod, H.; Gong, L.; Thorn, C.; Relling, M.V.; Klein, T.E.; et al. Etoposide pathway. *Pharmacogenet Genomics* **2009**, *19*, 552-553, doi:10.1097/FPC.0b013e32832e0e7f.
41. Product Information: FLUDARA IV injection, fludarabine phosphate IV injection. **2008**.
42. Product Information: fludarabine phosphate film coated oral tablets, oral tablets, fludarabine phosphate film coated oral tablets, oral tablets. **2008**.
43. Product Information: Adrucil, fluorouracil injection. **2003**.
44. Terret, C.; Erdociain, E.; Guimbaud, R.; Boisdron-Celle, M.; McLeod, H.L.; Fety-Deporte, R.; Lafont, T.; Gamelin, E.; Bugat, R.; Canal, P.; et al. Dose and time dependencies of 5-fluorouracil pharmacokinetics. *Clin Pharmacol Ther* **2000**, *68*, 270-279, doi:10.1067/mcp.2000.109352.
45. Product Information: Eulexin, flutamide. **2001**.
46. Katchen, B.; Buxbaum, S. Disposition of a new, nonsteroid, antiandrogen, *α,α,α*-trifluoro-2-methyl-4'-nitro-m-propionotoluidide (Flutamide), in men following a single oral 200 mg dose. *J Clin Endocrinol Metab* **1975**, *41*, 373-379, doi:10.1210/jcem-41-2-373.
47. Shet, M.S.; McPhaul, M.; Fisher, C.W.; Stallings, N.R.; Estabrook, R.W. Metabolism of the antiandrogenic drug (Flutamide) by human CYP1A2. *Drug Metab Dispos* **1997**, *25*, 1298-1303.
48. Product Information: GEMZAR intravenous injection, gemcitabine intravenous injection. **2013**.
49. Alvarellos, M.L.; Lamba, J.; Sangkuhl, K.; Thorn, C.F.; Wang, L.; Klein, D.J.; Altman, R.B.; Klein, T.E. PharmGKB summary: gemcitabine pathway. *Pharmacogenet Genomics* **2014**, *24*, 564-574, doi:10.1097/FPC.0000000000000086.
50. Product Information: Siklos oral tablets, hydroxyurea oral tablets. **2017**.
51. Product Information: HYDREA oral capsules, hydroxyurea oral capsules. **2015**.
52. Product Information: DROXIA oral capsules, hydroxyurea oral capsules. **2015**.
53. Gillies, H.C.; Herriott, D.; Liang, R.; Ohashi, K.; Rogers, H.J.; Harper, P.G. Pharmacokinetics of idarubicin (4-demethoxydaunorubicin; IMI-30; NSC 256439) following intravenous and oral administration in patients with advanced cancer. *Br J Clin Pharmacol* **1987**, *23*, 303-310, doi:10.1111/j.1365-2125.1987.tb03049.x.
54. Weiss, R.B.; Sarosy, G.; Clagett-Carr, K.; Russo, M.; Leyland-Jones, B. Anthracycline analogs: the past, present, and future. *Cancer Chemother Pharmacol* **1986**, *18*, 185-197, doi:10.1007/BF00273384.
55. Zanette, L.; Zucchetti, M.; Freshi, A.; Erranti, D.; Tirelli, U.; D'Incalci, M. Pharmacokinetics of 4-demethoxydaunorubicin in cancer patients. *Cancer Chemother Pharmacol* **1990**, *25*, 445-448, doi:10.1007/BF00686057.
56. Product Information: Ifex, ifosfamide. **1999**.
57. Fleming, R.A. An overview of cyclophosphamide and ifosfamide pharmacology. *Pharmacotherapy* **1997**, *17*, 146S-154S.
58. Kerbusch, T.; de Kraker, J.; Keizer, H.J.; van Putten, J.W.; Groen, H.J.; Jansen, R.L.; Schellens, J.H.; Beijnen, J.H. Clinical pharmacokinetics and pharmacodynamics of ifosfamide and its metabolites. *Clin Pharmacokinet* **2001**, *40*, 41-62, doi:10.2165/00003088-200140010-00004.
59. Kurowski, V.; Wagner, T. Urinary excretion of ifosfamide, 4-hydroxyifosfamide, 3- and 2-dechloroethylifosfamide, mesna, and dimesna in patients on fractionated intravenous ifosfamide and concomitant mesna therapy. *Cancer Chemother Pharmacol* **1997**, *39*, 431-439, doi:10.1007/s002800050594.
60. Product Information: GLEEVEC oral tablets, imatinib mesylate oral tablets. **2013**.
61. BC Cancer Agency Cancer Drug Manual. Imatinib. Available online: http://www.bccancer.bc.ca/drug-database-site/Drug%20Index/Imatinib_Monograph_1Mar2017.pdf (accessed on 16 August 2020).
62. Product Information: NIZORAL(R) oral tablets, ketoconazole oral tablets. **2013**.
63. Product Information: Femara tablets, letrozole tablets. **2004**.
64. Product Information: Femara, letrozole. **2003**.
65. BC Cancer Agency Cancer Drug Manual. Letrozole. Available online: http://www.bccancer.bc.ca/drug-database-site/Drug%20Index/Letrozole_monograph_1April2011.pdf (accessed on 16 August 2020).
66. Zittoun, J.; Tonelli, A.P.; Marquet, J.; De Gialluly, E.; Hancock, C.; Yacobi, A.; Johnson, J.B. Pharmacokinetic comparison of leucovorin and levoleucovorin. *Eur J Clin Pharmacol* **1993**, *44*, 569-573, doi:10.1007/BF02440861.
67. Product Information: leucovorin calcium IV injection, leucovorin calcium IV injection. **2007**.
68. Product Information: Megace, megestrol acetate tablets, USP. **2001**.
69. BC Cancer Agency Cancer Drug Manual. Megestrol. Available online: http://www.bccancer.bc.ca/drug-database-site/Drug%20Index/Megestrol_monograph_1June2013_formatted.pdf (accessed on 16 August 2020).
70. House, L.; Seminerio, M.J.; Mirkov, S.; Ramirez, J.; Skor, M.; Sachleben, J.R.; Isikbay, M.; Singhal, H.; Greene, G.L.; Vander Griend, D.; et al. Metabolism of megestrol acetate *in vitro* and the role of oxidative metabolites. *Xenobiotica* **2018**, *48*, 973-983, doi:10.1080/00498254.2017.1386335.
71. Product Information: ALKERAN oral tablets, melphalan oral tablets. **2011**.
72. Product Information: ALKERAN IV injection, melphalan HCl IV injection. **2011**.
73. Knoben, J.E.; Anderson, P.O. *Handbook of clinical drug data*, 6th ed.; Drug Intelligence Publications, Inc: Hamilton, IL, 1989.

74. Zimm, S.; Collins, J.M.; Riccardi, R.; O'Neill, D.; Narang, P.K.; Chabner, B.; Poplack, D.G. Variable bioavailability of oral mercaptopurine. Is maintenance chemotherapy in acute lymphoblastic leukemia being optimally delivered? *N Engl J Med* **1983**, *308*, 1005-1009, doi:10.1056/NEJM198304283081705.
75. Rundles, R.W.; Elion, G.B. Mercaptopurine "bioavailability". *N Engl J Med* **1984**, *310*, 929, doi:10.1056/NEJM198404053101421.
76. Product Information: PURIXAN oral suspension, mercaptopurine oral suspension. **2014**.
77. Product Information: PURINETHOL oral tablets, mercaptopurine oral tablets. **2011**.
78. Product Information: OTREXUP subcutaneous injection solution, methotrexate subcutaneous injection solution. **2013**.
79. Product Information: RASUVO subcutaneous injection, methotrexate subcutaneous injection. **2014**.
80. Product Information: XATMEP oral solution, methotrexate oral solution. **2017**.
81. Mikkelsen, T.S.; Thorn, C.F.; Yang, J.J.; Ulrich, C.M.; French, D.; Zaza, G.; Dunnenberger, H.M.; Marsh, S.; McLeod, H.L.; Giacomini, K.; et al. PharmGKB summary: methotrexate pathway. *Pharmacogenet Genomics* **2011**, *21*, 679-686, doi:10.1097/FPC.0b013e328343dd93.
82. Product Information: JELMYTO pyelocalyceal solution, mitomycin pyelocalyceal solution. **2020**.
83. Product Information: MITOMYCIN intravenous injection, mitomycin intravenous injection. **2009**.
84. Product Information: Lysodren, mitotane tablets. **1998**.
85. BC Cancer Agency Cancer Drug Manual. Mitotane. Available online: http://www.bccancer.bc.ca/drug-database-site/Drug%20Index/Mitotane_monograph_1June2013.pdf (accessed on 16 August 2020).
86. Product Information: Novantrone, mitoxantrone. **2000**.
87. Ehninger, G.; Proksch, B.; Hartmann, F.; Gartner, H.V.; Wilms, K. Mitoxantrone metabolism in the isolated perfused rat liver. *Cancer Chemother Pharmacol* **1984**, *12*, 50-52, doi:10.1007/BF00255910.
88. Koeller, J.; Eble, M. Mitoxantrone: a novel anthracycline derivative. *Clin Pharm* **1988**, *7*, 574-581.
89. Product Information: TASIGNA oral capsules, nilotinib oral capsules. **2007**.
90. BC Cancer Agency Cancer Drug Manual. Nilotinib Available online: http://www.bccancer.bc.ca/drug-database-site/Drug%20Index/Nilotinib_monograph_1Mar2017.pdf (accessed on 16 August 2020).
91. Product Information: TASIGNA oral capsules, nilotinib oral capsules. **2018**.
92. Product Information: Taxol, paclitaxel. **2000**.
93. BC Cancer Agency Cancer Drug Manual. Paclitaxel. Available online: http://www.bccancer.bc.ca/drug-database-site/Drug%20Index/Paclitaxel_monograph.pdf (accessed on 16 August 2020).
94. Product Information: Matulane, procarbazine hydrochloride capsules. **2002**.
95. Chabner, B.A.; Myers, C.E.; Oliverio, V.T. Clinical pharmacology of anticancer drugs. *Semin Oncol* **1977**, *4*, 165-191.
96. Schwartz, D.E.; Bollag, W.; Obrecht, P. Distribution and excretion studies of procarbazine in animals and man. *Arzneimittelforschung* **1967**, *17*, 1389-1393.
97. Spivack, S.D. Drugs 5 years later: procarbazine. *Ann Intern Med* **1974**, *81*, 795-800, doi:10.7326/0003-4819-81-6-795.
98. Product Information: Nolvadex, tamoxifen citrate. **2003**.
99. BC Cancer Drug Manual. Tamoxifen. Available online: http://www.bccancer.bc.ca/drug-database-site/Drug%20Index/Tamoxifen_monograph.pdf (accessed on 18 May 2021).
100. Product Information: SOLTAMOX oral solution, tamoxifen citrate oral solution. **2018**.
101. Heel, R.C.; Brogden, R.N.; Speight, T.M.; Avery, G.S. Tamoxifen: a review of its pharmacological properties and therapeutic use in the treatment of breast cancer. *Drugs* **1978**, *16*, 1-24, doi:10.2165/00003495-197816010-00001.
102. Klein, D.J.; Thorn, C.F.; Desta, Z.; Flockhart, D.A.; Altman, R.B.; Klein, T.E. PharmGKB summary: tamoxifen pathway, pharmacokinetics. *Pharmacogenet Genomics* **2013**, *23*, 643-647, doi:10.1097/FPC.0b013e3283656bc1.
103. Sanchez-Spitman, A.; Dezentje, V.; Swen, J.; Moes, D.; Bohringer, S.; Batman, E.; van Druten, E.; Smorenburg, C.; van Bochove, A.; Zeillemaker, A.; et al. Tamoxifen pharmacogenetics and metabolism: results from the prospective CYPTAM study. *J Clin Oncol* **2019**, *37*, 636-646, doi:10.1200/JCO.18.00307.
104. Product Information: TABLOID oral tablets, thioguanine oral tablets **2018**.
105. Zaza, G.; Cheok, M.; Krynetskaia, N.; Thorn, C.; Stocco, G.; Hebert, J.M.; McLeod, H.; Weinshilboum, R.M.; Relling, M.V.; Evans, W.E.; et al. Thiopurine pathway. *Pharmacogenet Genomics* **2010**, *20*, 573-574, doi:10.1097/FPC.0b013e328334338f.
106. Product Information: HYCAMTIN(R) oral capsules, topotecan oral capsules. **2007**.
107. Product Information: HYCAMTIN(R) oral capsules, topotecan oral capsules. **2018**.
108. Product Information: VESANOID oral capsules, tretinoin oral capsules. **2008**.
109. BC Cancer Agency Cancer Drug Manual. Tretinoin. Available online: http://www.bccancer.bc.ca/drug-database-site/Drug%20Index/Tretinoin_monograph_1Feb2014.pdf (accessed on 16 August 2020).
110. Zhou-Pan, X.R.; Seree, E.; Zhou, X.J.; Placidi, M.; Maurel, P.; Barra, Y.; Rahmani, R. Involvement of human liver cytochrome P450 3A in vinblastine metabolism: drug interactions. *Cancer Res* **1993**, *53*, 5121-5126.
111. Product Information: vincristine sulfate IV Injection, vincristine sulfate IV Injection. **2007**.
112. Jehl, F.; Quoix, E.; Leveque, D.; Pauli, G.; Breillout, F.; Krikorian, A.; Monteil, H. Pharmacokinetic and preliminary metabolic fate of navelbine in humans as determined by high performance liquid chromatography. *Cancer Res* **1991**, *51*, 2073-2076.
113. Funde, S.G. Phytochemicals evaluation, anticancer, antioxidant and antimicrobial activity of *Acorus calamus* different solvent extracts. *J Chem Pharm Res* **2015**, *7*, 495-504.
114. Liu, L.; Wang, J.; Shi, L.; Zhang, W.; Du, X.; Wang, Z.; Zhang, Y. β-Asarone induces senescence in colorectal cancer cells by inducing lamin B1 expression. *Phytomedicine* **2013**, *20*, 512-520, doi:10.1016/j.phymed.2012.12.008.

115. Melek, F.R.; Miyase, T.; Ghaly, N.S.; Nabil, M. Triterpenoid saponins with *N*-acetyl sugar from the bark of *Albizia procera*. *Phytochemistry* **2007**, *68*, 1261-1266, doi:10.1016/j.phytochem.2007.02.023.
116. Li, W.; Tian, H.; Li, L.; Li, S.; Yue, W.; Chen, Z.; Qi, L.; Hu, W.; Zhu, Y.; Hao, B.; et al. Diallyl trisulfide induces apoptosis and inhibits proliferation of A549 cells *in vitro* and *in vivo*. *Acta Biochim Biophys Sin (Shanghai)* **2012**, *44*, 577-583, doi:10.1093/abbs/gms033.
117. Lai, Y.H.; Yu, S.L.; Chen, H.Y.; Wang, C.C.; Chen, H.W.; Chen, J.J. The HLJ1-targeting drug screening identified Chinese herb andrographolide that can suppress tumour growth and invasion in non-small-cell lung cancer. *Carcinogenesis* **2013**, *34*, 1069-1080, doi:10.1093/carcin/bgt005.
118. Sheeja, K.; Guruvayoorappan, C.; Kuttan, G. Antiangiogenic activity of *Andrographis paniculata* extract and andrographolide. *Int Immunopharmacol* **2007**, *7*, 211-221, doi:10.1016/j.intimp.2006.10.002.
119. Choochuay, K.; Chunhacha, P.; Pongrakhananon, V.; Luechapudiporn, R.; Chanvorachote, P. Imperatorin sensitizes anoikis and inhibits anchorage-independent growth of lung cancer cells. *J Nat Med* **2013**, *67*, 599-606, doi:10.1007/s11418-012-0719-y.
120. Luo, K.W.; Sun, J.G.; Chan, J.Y.; Yang, L.; Wu, S.H.; Fung, K.P.; Liu, F.Y. Anticancer effects of imperatorin isolated from *Angelica dahurica*: induction of apoptosis in HepG2 cells through both death-receptor- and mitochondria-mediated pathways. *Chemotherapy* **2011**, *57*, 449-459, doi:10.1159/000331641.
121. Tsai, N.M.; Chen, Y.L.; Lee, C.C.; Lin, P.C.; Cheng, Y.L.; Chang, W.L.; Lin, S.Z.; Harn, H.J. The natural compound *n*-butylidenephthalide derived from *Angelica sinensis* inhibits malignant brain tumor growth *in vitro* and *in vivo*. *J Neurochem* **2006**, *99*, 1251-1262, doi:10.1111/j.1471-4159.2006.04151.x.
122. Chen, Q.C.; Lee, J.; Jin, W.; Youn, U.; Kim, H.; Lee, I.S.; Zhang, X.; Song, K.; Seong, Y.; Bae, K. Cytotoxic constituents from *Angelicae sinensis* radix. *Arch Pharm Res* **2007**, *30*, 565-569, doi:10.1007/BF02977650.
123. Mahavorasirikul, W.; Viyanant, V.; Chaijaroenkul, W.; Itharat, A.; Na-Bangchang, K. Cytotoxic activity of Thai medicinal plants against human cholangiocarcinoma, laryngeal and hepatocarcinoma cells *in vitro*. *BMC Complement Altern Med* **2010**, *10*, 55, doi:10.1186/1472-6882-10-55.
124. Plengsuriyakarn, T.; Viyanant, V.; Eursitthichai, V.; Picha, P.; Kupradinun, P.; Itharat, A.; Na-Bangchang, K. Anticancer activities against cholangiocarcinoma, toxicity and pharmacological activities of Thai medicinal plants in animal models. *BMC Complement Altern Med* **2012**, *12*, 23, doi:10.1186/1472-6882-12-23.
125. Park, J.-H.; Jeon, G.-I.; Kim, J.-M.; Park, E. Antioxidant activity and antiproliferative action of methanol extracts of 4 different colored bell peppers (*Capsicum annuum* l.). *Food Sci Biotechnol* **2012**, *21*, 543-550, doi:10.1007/s10068-012-0069-2.
126. Lee, S.H.; Richardson, R.L.; Dashwood, R.H.; Baek, S.J. Capsaicin represses transcriptional activity of β-catenin in human colorectal cancer cells. *J Nutr Biochem* **2012**, *23*, 646-655, doi:10.1016/j.jnutbio.2011.03.009.
127. Fernández-Bedmar, Z.; Alonso-Moraga, A. *In vivo* and *in vitro* evaluation for nutraceutical purposes of capsaicin, capsanthin, lutein and four pepper varieties. *Food Chem Toxicol* **2016**, *98*, 89-99, doi:10.1016/j.fct.2016.10.011.
128. Jeon, J.H.; Choi, Y.J.; Han, I.H.; Choi, B.K.; Cha, S.H.; Cho, W.H. Capsaicin-induced apoptosis in the human glioblastoma U87MG cells via p-38 MAPK and Bcl-2/Bax signaling pathway. *Mol Cell Toxicol* **2012**, *8*, 69-76, doi:10.1007/s13273-012-0009-5.
129. Lin, C.H.; Lu, W.C.; Wang, C.W.; Chan, Y.C.; Chen, M.K. Capsaicin induces cell cycle arrest and apoptosis in human KB cancer cells. *BMC Complement Altern Med* **2013**, *13*, 46, doi:10.1186/1472-6882-13-46.
130. Bogucka-Kocka, A.; Smolarz, H.D.; Kocki, J. Apoptotic activities of ethanol extracts from some Apiaceae on human leukaemia cell lines. *Fitoterapia* **2008**, *79*, 487-497, doi:10.1016/j.fitote.2008.07.002.
131. Kamaleeswari, M.; Nalini, N. Dose-response efficacy of caraway (*Carum carvi* L.) on tissue lipid peroxidation and antioxidant profile in rat colon carcinogenesis. *J Pharm Pharmacol* **2006**, *58*, 1121-1130, doi:10.1211/jpp.58.8.0014.
132. Kamaleeswari, M.; Deeptha, K.; Sengottuvelan, M.; Nalini, N. Effect of dietary caraway (*Carum carvi* L.) on aberrant crypt foci development, fecal steroids, and intestinal alkaline phosphatase activities in 1,2-dimethylhydrazine-induced colon carcinogenesis. *Toxicol Appl Pharmacol* **2006**, *214*, 290-296, doi:10.1016/j.taap.2006.01.001.
133. Aydin, E.; Turkez, H.; Keles, M.S. Potential anticancer activity of carvone in N2a neuroblastoma cell line. *Toxicol Ind Health* **2015**, *31*, 764-772, doi:10.1177/0748233713484660.
134. Babykutty, S.; Padikkala, J.; Sathiadevan, P.P.; Vijayakurup, V.; Azis, T.K.; Srinivas, P.; Gopala, S. Apoptosis induction of *Centella asiatica* on human breast cancer cells. *Afr J Tradit Complement Altern Med* **2008**, *6*, 9-16, doi:10.4314/ajtcam.v6i1.57068.
135. Tang, X.L.; Yang, X.Y.; Jung, H.J.; Kim, S.Y.; Jung, S.Y.; Choi, D.Y.; Park, W.C.; Park, H. Asiatic acid induces colon cancer cell growth inhibition and apoptosis through mitochondrial death cascade. *Biol Pharm Bull* **2009**, *32*, 1399-1405, doi:10.1248/bpb.32.1399.
136. Somwong, P.; Suttisri, R. Cytotoxic activity of the chemical constituents of *Clerodendrum indicum* and *Clerodendrum villosum* roots. *J Integr Med* **2018**, *16*, 57-61, doi:10.1016/j.joim.2017.12.004.
137. Gagandeep; Dhanalakshmi, S.; Mendiz, E.; Rao, A.R.; Kale, R.K. Chemopreventive effects of *Cuminum cyminum* in chemically induced forestomach and uterine cervix tumors in murine model systems. *Nutr Cancer* **2003**, *47*, 171-180, doi:10.1207/s15327914nc4702_10.
138. Zhu, J.Y.; Yang, X.; Chen, Y.; Jiang, Y.; Wang, S.J.; Li, Y.; Wang, X.Q.; Meng, Y.; Zhu, M.M.; Ma, X.; et al. Curcumin suppresses lung cancer stem cells via inhibiting Wnt/β-catenin and sonic hedgehog pathways. *Phytother Res* **2017**, *31*, 680-688, doi:10.1002/ptr.5791.
139. Mishra, D.; Singh, S.; Narayan, G. Curcumin induces apoptosis in pre-B acute lymphoblastic leukemia cell lines via PARP-1 cleavage. *Asian Pac J Cancer Prev* **2016**, *17*, 3865-3869.

140. Yue, G.G.; Chan, B.C.; Hon, P.M.; Lee, M.Y.; Fung, K.P.; Leung, P.C.; Lau, C.B. Evaluation of *in vitro* anti-proliferative and immunomodulatory activities of compounds isolated from *Curcuma longa*. *Food Chem Toxicol* **2010**, *48*, 2011-2020, doi:10.1016/j.fct.2010.04.039.
141. Appadath Beeran, A.; Maliyakkal, N.; Rao, C.M.; Udupa, N. The enriched fraction of *Vernonia cinerea* L. induces apoptosis and inhibits multi-drug resistance transporters in human epithelial cancer cells. *J Ethnopharmacol* **2014**, *158*, 33-42, doi:10.1016/j.jep.2014.10.010.
142. Khay, M.; Toeng, P.; Mahiou-Leddet, V.; Mabrouki, F.; Sothea, K.; Ollivier, E.; Elias, R.; Bun, S.-S. HPLC analysis and cytotoxic activity of *Vernonia cinerea*. *Nat Prod Commun* **2012**, *7*, 1259-1262, doi:10.1177/1934578X1200701001.
143. Kuo, Y.-H.; Kuo, Y.-J.; Yu, A.-S.; Wu, M.-D.; Ong, C.-W.; Yang Kuo, L.-M.; Huang, J.-T.; Chen, C.-F.; Li, S.-Y. Two novel sesquiterpene lactones, cytotoxic vernolide-A and -B, from *Vernonia cinerea*. *Chem Pharm Bull* **2003**, *51*, 425-426, doi:10.1248/cpb.51.425.
144. Pratheeshkumar, P.; Kuttan, G. Antimetastatic potential of vernolide-A, a sesquiterpenoid from *Vernonia cinerea* L. *Hum Exp Toxicol* **2012**, *31*, 66-80, doi:10.1177/0960327111414279.
145. Pratheeshkumar, P.; Kuttan, G. Modulation of cytotoxic T lymphocyte, natural killer cell, antibody-dependent cellular cytotoxicity, and antibody-dependent complement-mediated cytotoxicity by *Vernonia cinerea* L. and vernolide-A in BALB/c mice via enhanced production of cytokines IL-2 and IFN-$\gamma$. *Immunopharmacol Immunotoxicol* **2012**, *34*, 46-55, doi:10.3109/08923973.2011.574703.
146. Ueda, J.Y.; Tezuka, Y.; Banskota, A.H.; Le Tran, Q.; Tran, Q.K.; Harimaya, Y.; Saiki, I.; Kadota, S. Antiproliferative activity of Vietnamese medicinal plants. *Biol Pharm Bull* **2002**, *25*, 753-760, doi:10.1248/bpb.25.753.
147. Mahfudh, N.; Lope Pihie, A.H. Eurycomanone induces apoptosis through the up-regulation of p53 in human cervical carcinoma cells. *Cancer Mol* **2008**, *4*, 109-115.
148. Zakaria, Y.; Rahmat, A.; Pihie, A.H.L.; Abdullah, N.R.; Houghton, P.J. Eurycomanone induce apoptosis in HepG2 cells via up-regulation of p53. *Cancer Cell Int* **2009**, *9*, 16, doi:10.1186/1475-2867-9-16.
149. Li, R.W.; Leach, D.N.; Myers, S.P.; Lin, G.D.; Leach, G.J.; Waterman, P.G. A new anti-inflammatory glucoside from *Ficus racemosa* L. *Planta Med* **2004**, *70*, 421-426.
150. Ahmed, F.; Urooj, A.; Karim, A.A. Protective effects of *Ficus racemosa* stem bark against doxorubucin-induced renal and testicular toxicity. *Pharmacogn Mag* **2013**, *9*, 130-134, doi:10.4103/0973-1296.111265.
151. Singh, B.; Kale, R.K. Chemomodulatory action of *Foeniculum vulgare* (Fennel) on skin and forestomach papillomagenesis, enzymes associated with xenobiotic metabolism and antioxidant status in murine model system. *Food Chem Toxicol* **2008**, *46*, 3842-3850, doi:10.1016/j.fct.2008.10.008.
152. Choo, E.J.; Rhee, Y.H.; Jeong, S.J.; Lee, H.J.; Kim, H.S.; Ko, H.S.; Kim, J.H.; Kwon, T.R.; Jung, J.H.; Kim, J.H.; et al. Anethole exerts antimetatstaic activity via inhibition of matrix metalloproteinase 2/9 and akt/mitogen-activated kinase/nuclear factor kappa B signaling pathways. *Biol Pharm Bull* **2011**, *34*, 41-46, doi:10.1248/bpb.34.41.
153. Cheng, T.-C.; Lu, J.-F.; Wang, J.-S.; Lin, L.-J.; Kuo, H.-I.; Chen, B.-H. Antiproliferation effect and apoptosis mechanism of prostate cancer cell PC-3 by flavonoids and saponins prepared from *Gynostemma pentaphyllum*. *J Agric Food Chem* **2011**, *59*, 11319-11329, doi:10.1021/jf2018758.
154. Li, Y.; Lin, W.; Huang, J.; Xie, Y.; Ma, W. Anti-cancer effects of *Gynostemma pentaphyllum* (Thunb.) Makino (Jiaogulan). *Chin Med* **2016**, *11*, 43, doi:10.1186/s13020-016-0114-9.
155. Chen, J.C.; Lu, K.W.; Tsai, M.L.; Hsu, S.C.; Kuo, C.L.; Yang, J.S.; Hsia, T.C.; Yu, C.S.; Chou, S.T.; Kao, M.C.; et al. Gypenosides induced G0/G1 arrest via CHk2 and apoptosis through endoplasmic reticulum stress and mitochondria-dependent pathways in human tongue cancer SCC-4 cells. *Oral Oncol* **2009**, *45*, 273-283, doi:10.1016/j.oraloncology.2008.05.012.
156. Lin, J.-J.; Hsu, H.-Y.; Yang, J.-S.; Lu, K.-W.; Wu, R.S.-C.; Wu, K.-C.; Lai, T.-Y.; Chen, P.-Y.; Ma, C.-Y.; Wood, W.G.; et al. Molecular evidence of anti-leukemia activity of gypenosides on human myeloid leukemia HL-60 cells *in vitro* and *in vivo* using a HL-60 cells murine xenograft model. *Phytomedicine* **2011**, *18*, 1075-1085, doi:10.1016/j.phymed.2011.03.009.
157. Zheng, K.; Liao, C.; Li, Y.; Fan, X.; Fan, L.; Xu, H.; Kang, Q.; Zeng, Y.; Wu, X.; Wu, H.; et al. Gypenoside L, isolated from *Gynostemma pentaphyllum*, induces cytoplasmic vacuolation death in hepatocellular carcinoma cells through reactive-oxygen-species-mediated unfolded protein response. *J Agric Food Chem* **2016**, *64*, 1702-1711, doi:10.1021/acs.jafc.5b05668.
158. Yin, S.; Chen, X.; Su, Z.-S.; Yang, S.-P.; Fan, C.-Q.; Ding, J.; Yue, J.-M. Harrisotones A–E, five novel prenylated polyketides with a rare spirocyclic skeleton from *Harrisonia perforata*. *Tetrahedron* **2009**, *65*, 1147-1152, doi:10.1016/j.tet.2008.11.068.
159. Ru, P.; Steele, R.; Nerurkar, P.V.; Phillips, N.; Ray, R.B. Bitter melon extract impairs prostate cancer cell-cycle progression and delays prostatic intraepithelial neoplasia in TRAMP model. *Cancer Prev Res (Phila)* **2011**, *4*, 2122-2130, doi:10.1158/1940-6207.CAPR-11-0376.
160. Sreelatha, S.; Jeyachitra, A.; Padma, P.R. Antiproliferation and induction of apoptosis by *Moringa oleifera* leaf extract on human cancer cells. *Food Chem Toxicol* **2011**, *49*, 1270-1275, doi:10.1016/j.fct.2011.03.006.
161. Koontongkaew, S.; Suriyawong, K.; Siengboon, W. Effects of *Murdannia loriformis* extracts on cancer cell growth. Pathumthani, Thailand, July 2009.
162. Jiratchariyakul, W.; Okabe, H.; Moongkarndi, P.; Frahm, A. Cytotoxic glycoshingolipid from *Murdannia loriformis* (Hassk.) Rolla Rao et Kammathy. *Thai J Phytopharmacy* **1998**, *5*, 10-20.
163. Chaudhary, S.; Chandrashekar, K.S.; Pai, K.S.; Setty, M.M.; Devkar, R.A.; Reddy, N.D.; Shoja, M.H. Evaluation of antioxidant and anticancer activity of extract and fractions of *Nardostachys jatamansi* DC in breast carcinoma. *BMC Complement Altern Med* **2015**, *15*, 50, doi:10.1186/s12906-015-0563-1.

164. Chang, C.H.; Ou, T.T.; Yang, M.Y.; Huang, C.C.; Wang, C.J. *Nelumbo nucifera* Gaertn leaves extract inhibits the angiogenesis and metastasis of breast cancer cells by downregulation connective tissue growth factor (CTGF) mediated PI3K/AKT/ERK signaling. *J Ethnopharmacol* **2016**, *188*, 111-122, doi:10.1016/j.jep.2016.05.012.
165. Poornima, P.; Weng, C.F.; Padma, V.V. Neferine, an alkaloid from lotus seed embryo, inhibits human lung cancer cell growth by MAPK activation and cell cycle arrest. *Biofactors* **2014**, *40*, 121-131, doi:10.1002/biof.1115.
166. ElKhoely, A.; Hafez, H.F.; Ashmawy, A.M.; Badary, O.; Abdelaziz, A.; Mostafa, A.; Shouman, S.A. Chemopreventive and therapeutic potentials of thymoquinone in HepG2 cells: mechanistic perspectives. *J Nat Med* **2015**, *69*, 313-323, doi:10.1007/s11418-015-0895-7.
167. Xu, D.; Ma, Y.; Zhao, B.; Li, S.; Zhang, Y.; Pan, S.; Wu, Y.; Wang, J.; Wang, D.; Pan, H.; et al. Thymoquinone induces G2/M arrest, inactivates PI3K/Akt and nuclear factor-kappaB pathways in human cholangiocarcinomas both *in vitro* and *in vivo*. *Oncol Rep* **2014**, *31*, 2063-2070, doi:10.3892/or.2014.3059.
168. Chu, S.C.; Hsieh, Y.S.; Yu, C.C.; Lai, Y.Y.; Chen, P.N. Thymoquinone induces cell death in human squamous carcinoma cells via caspase activation-dependent apoptosis and LC3-II activation-dependent autophagy. *PLoS One* **2014**, *9*, e101579, doi:10.1371/journal.pone.0101579.
169. Khalife, R.; Hodroj, M.H.; Fakhoury, R.; Rizk, S. Thymoquinone from *Nigella sativa* seeds promotes the antitumor activity of noncytotoxic doses of topotecan in human colorectal cancer cells *in vitro*. *Planta Med* **2016**, *82*, 312-321, doi:10.1055/s-0035-1558289.
170. Rajani, P.; Kotaiah, R.; Sekar, C. Evaluation of antioxidant and anticancer activities of *Orthosiphon aristatus* (Blume). *Int J Res Pharm Sci* **2015**, *6*, 193-198.
171. Dolečková, I.; Rárová, L.; Grúz, J.; Vondrusová, M.; Strnad, M.; Kryštof, V. Antiproliferative and antiangiogenic effects of flavone eupatorin, an active constituent of chloroform extract of *Orthosiphon stamineus* leaves. *Fitoterapia* **2012**, *83*, 1000-1007, doi:10.1016/j.fitote.2012.06.002.
172. Liu, X.; Zhao, M.; Wu, K.; Chai, X.; Yu, H.; Tao, Z.; Wang, J. Immunomodulatory and anticancer activities of phenolics from emblica fruit (*Phyllanthus emblica* L.). *Food Chem* **2012**, *131*, 685-690, doi:10.1016/j.foodchem.2011.09.063.
173. Kumar, S.; Pathania, A.S.; Satti, N.K.; Dutt, P.; Sharma, N.; Mallik, F.A.; Ali, A. Synthetic modification of hydroxychavicol by Mannich reaction and alkyne-azide cycloaddition derivatives depicting cytotoxic potential. *Eur J Med Chem* **2015**, *92*, 236-245, doi:10.1016/j.ejmech.2014.12.047.
174. Gundala, S.R.; Yang, C.; Mukkavilli, R.; Paranjpe, R.; Brahmbhatt, M.; Pannu, V.; Cheng, A.; Reid, M.D.; Aneja, R. Hydroxychavicol, a betel leaf component, inhibits prostate cancer through ROS-driven DNA damage and apoptosis. *Toxicol Appl Pharmacol* **2014**, *280*, 86-96, doi:10.1016/j.taap.2014.07.012.
175. de Souza Grinevicius, V.M.; Kviecinski, M.R.; Santos Mota, N.S.; Ourique, F.; Porfirio Will Castro, L.S.; Andreguetti, R.R.; Gomes Correia, J.F.; Filho, D.W.; Pich, C.T.; Pedrosa, R.C. *Piper nigrum* ethanolic extract rich in piperamides causes ROS overproduction, oxidative damage in DNA leading to cell cycle arrest and apoptosis in cancer cells. *J Ethnopharmacol* **2016**, *189*, 139-147, doi:10.1016/j.jep.2016.05.020.
176. Gunasekaran, V.; Elangovan, K.; Niranjali Devaraj, S. Targeting hepatocellular carcinoma with piperine by radical-mediated mitochondrial pathway of apoptosis: An *in vitro* and *in vivo* study. *Food Chem Toxicol* **2017**, *105*, 106-118, doi:10.1016/j.fct.2017.03.029.
177. Do, M.T.; Kim, H.G.; Choi, J.H.; Khanal, T.; Park, B.H.; Tran, T.P.; Jeong, T.C.; Jeong, H.G. Antitumor efficacy of piperine in the treatment of human HER2-overexpressing breast cancer cells. *Food Chem* **2013**, *141*, 2591-2599, doi:10.1016/j.foodchem.2013.04.125.
178. Zainal Ariffin, S.H.; Wan Omar, W.H.; Zainal Ariffin, Z.; Safian, M.F.; Senafi, S.; Megat Abdul Wahab, R. Intrinsic anticarcinogenic effects of *Piper sarmentosum* ethanolic extract on a human hepatoma cell line. *Cancer Cell Int* **2009**, *9*, 6, doi:10.1186/1475-2867-9-6.
179. Sohn, V.R.; Giros, A.; Xicola, R.M.; Fluvia, L.; Grzybowski, M.; Anguera, A.; Llor, X. Stool-fermented *Plantago ovata* husk induces apoptosis in colorectal cancer cells independently of molecular phenotype. *Br J Nutr* **2012**, *107*, 1591-1602, doi:10.1017/S0007114511004910.
180. Olarte, E.I.; Herrera, A.A.; Villasenor, I.M.; Jacinto, S.D. *In vitro* antitumor properties of an isolate from leaves of *Cassia alata* L. *Asian Pac J Cancer Prev* **2013**, *14*, 3191-3196.
181. Kimura, Y.; Sumiyoshi, M.; Taniguchi, M.; Baba, K. Antitumor and antimetastatic actions of anthrone-C-glucoside, cassialoin isolated from *Cassia garrettiana* heartwood in colon 26-bearing mice. *Cancer Sci* **2008**, *99*, 2336-2348, doi:10.1111/j.1349-7006.2008.00938.x.
182. Sangmalee, S.; Laorpaksa, A.; Sritularak, B.; Sukrong, S. Bioassay-guided isolation of two flavonoids from *Derris scandens* with topoisomerase II poison activity. *Biol Pharm Bull* **2016**, *39*, 631-635, doi:10.1248/bpb.b15-00767.
183. Chen, Y.-X.; Tong, J.; Ge, L.-L.; Ma, B.-X.; He, J.-S.; Wang, Y.-W. Ethyl acetate fraction of *Terminalia bellirica* fruit inhibits rat hepatic stellate cell proliferation and induces apoptosis. *Ind Crops Prod* **2015**, *76*, 364-373, doi:10.1016/j.indcrop.2015.07.007.
184. Das, N.D.; Jung, K.H.; Park, J.H.; Mondol, M.A.M.; Shin, H.J.; Lee, H.-S.; Park, K.S.; Choi, M.R.; Kim, K.S.; Kim, M.S.; et al. *Terminalia chebula* extract acts as a potential NF-κB inhibitor in human lymphoblastic T cells. *Phytother Res* **2011**, *25*, 927-934, doi:10.1002/ptr.3398.
185. Kumar, N.; Gangappa, D.; Gupta, G.; Karnati, R. Chebulagic acid from *Terminalia chebula* causes G1 arrest, inhibits NFκB and induces apoptosis in retinoblastoma cells. *BMC Complement Altern Med* **2014**, *14*, 319, doi:10.1186/1472-6882-14-319.
186. Kapoor, V.; Aggarwal, S.; Das, S.N. 6-Gingerol mediates its anti tumor activities in human oral and cervical cancer cell lines through apoptosis and cell cycle arrest. *Phytother Res* **2016**, *30*, 588-595, doi:10.1002/ptr.5561.
